# Genetic diversity of common beans (*Phaseolus vulgaris* L.) and runner beans (*Phaseolus coccineus* L.): a case study from *in-situ* preserved landraces in Northern Italy

**DOI:** 10.3389/fpls.2026.1838084

**Published:** 2026-07-17

**Authors:** Alessandra Lezzi, Elena Petretto, Alessandra Lanubile, Francesco Ferrari, Graziano Rossi, Adriano Marocco, Lorenzo Stagnati, Matteo Busconi

**Affiliations:** 1 Dipartimento di Scienze delle Produzioni Vegetali Sostenibili DI.PRO.VE.S, Università Cattolica del Sacro Cuore, Piacenza, Italy; 2 Centro di Ricerca sulla Biodiversità e sul DNA antico (BioDNA), Università Cattolica del Sacro Cuore, Piacenza, Italy; 3 Dipartimento di Scienze della Terra e dell'Ambiente DSTA, Università degli Studi di Pavia, Pavia, Italy

**Keywords:** genetic characterization, germplasm, Italian bean landraces, *Phaseolus coccineus*, *Phaseolus vulgaris*

## Abstract

**Introduction:**

In Lombardia region of Italy, several collections of *Phaseolus vulgaris* and *Phaseolus coccineus* landraces are conserved in specialized repositories (germplasm banks), yet their morphological characterization and genetic potential remain poorly documented. Given the importance of common (*P. vulgaris)* and runner (*P. coccineus*) bean landraces as genetic resources for biodiversity conservation, resilience to biotic and abiotic stresses, and crop improvement, this study aimed to analyse threatened bean landraces from Lombardia that are at risk of extinction and genetic erosion.

**Methods:**

A total of eight common bean and seven runner bean accessions were sampled, morphologically characterized *in-situ*, and genetically analysed using a genotyping-by-sequencing approach to assess genetic diversity and population structure. The results showed no clear clustering of landraces based on geographic origin for either species.

**Results:**

Common bean landraces exhibited distinct genetic identities, characterized by high inbreeding coefficients and low observed heterozygosity, consistent with their predominantly autogamous mating system, and strong genetic differentiation was detected among landraces. In contrast, runner bean landraces showed clustering of individuals within populations, higher observed heterozygosity, low inbreeding coefficients, and limited genetic differentiation, reflecting their allogamous pollination system.

**Discussion:**

These findings highlight contrasting genetic structures between the two species and provide a foundation for the conservation and valorisation of Lombardia bean landraces.

## 1 Introduction

Common (*Phaseolus vulgaris* L.) and runner beans (*Phaseolus coccineus* L.) are among the most important species in the genus *Phaseolus* within the *Fabaceae* family (Torheim and Fadnes, 2024; Vargas-Vázquez et al., 2024). These beans are annual herbaceous plants cultivated worldwide, originally coming from Central America, for the consumption of their seeds, as dry, fresh grain (shell beans), or immature pods (snap beans). The plant has rapid growth and a relatively short reproductive cycle, high variability in seed shape, colour and size, provides yield that can be easily stocked for long time, and it adapts well to various climates thanks to a great variability in plants’ architecture; all these characteristics makes this legume crop extremely important in different agroecosystems as an integral part of the diet (Caproni et al., 2020; Geleta et al., 2024).

It can be said that beans are an essential crop if we aim to make global agriculture more sustainable promoting symbiotic nitrogen fixation, increasing soil fertility and structure, as well as promoting biodiversity in our agroecosystems (Islam et al., 2024; Lisciani et al., 2024; Geleta et al., 2024; Mauceri et al., 2025). Regarding the human and animal diets, legumes are an excellent source of B-group vitamins and vitamin C, minerals and they are rich in linoleic and oleic acid, and bioactive compounds with beneficial functional properties (Lisciani et al., 2024). Legumes are also an excellent source of protein and can replace the excessive consumption of meat (Willett et al., 2019; Torheim and Fadnes, 2024).

Although beans are suitable for food security and they could enhance agricultural sustainability, still limited research on their genetic and limited improvement on cultivated variety has been done making this crop uncompetitive compared to others, such as maize and soybean, and sensitive to climate change (Caproni et al., 2020; Geleta et al., 2024). Climate-induced moisture stress, diseases, and insect pests pose significant challenges to its productivity. Furthermore, breeding programs focused on increasing yield have reduced the quality of today cultivated varieties (Márquez et al., 2024). In this context, to enhance stress resistance and improve the nutritional quality of cultivated varieties, it is important to explore the genetic diversity of bean landraces (Villa et al., 2005; Márquez et al., 2024). The long tradition of Phaseolus cultivation in Italy, following its introduction into Europe in the 16th century (Angioi et al., 2010), has led to the development of numerous locally adapted landraces, particularly in marginal environments where microclimatic conditions shaped distinct ecotypes with unique morpho-agronomic and nutritional traits (Venora et al., 2009). Landraces are genetically heterogeneous populations shaped by long-term environmental adaptation and farmer selection, which distinguishes them from modern cultivars (Villa et al., 2005; Raggi et al., 2019; Sangiorgio et al., 2021; Almeida et al., 2024). Over time, they have been empirically selected for traits such as yield stability, adaptation, taste, and tolerance to biotic and abiotic stresses (Aquino-Bolaños et al., 2021; Lezzi et al., 2026). As a result, they represent a broad and largely untapped genetic pool, providing valuable alleles for crop improvement and breeding programs aimed at resilience and nutritional quality (Celmeli et al., 2018; Canella et al., 2022; Caproni et al., 2020; Aquino-Bolaños et al., 2021). Beyond their agronomic value, landraces contribute to the conservation of agrobiodiversity and local knowledge systems and are typically maintained in low-input farming systems for self-consumption or niche markets (Piergiovanni and Lioi, 2010; Lioi et al., 2012; Simioni et al., 2024). However, their progressive replacement by modern cultivars is leading to genetic erosion, highlighting the importance of their characterization and valorisation for sustainable agriculture and food security.

In Italy, common beans have a long agricultural tradition, and this allowed the evolution of many landraces specifically adapted to restricted areas (Piergiovanni and Lioi, 2010). At the beginning of the 20th century, Comes (1910) reported 472 common bean varieties, and this richness has since been confirmed by several authors. According to Piergiovanni and Lioi (2010), the CNR Institute of Plant Genetics (IGV, Bari, Italy) preserves 1,500 accessions of Italian germplasm. Additional material is conserved at the Leibniz Institute of Plant Genetics and Crop Plant Research (IPK, Germany), as well as in several smaller collections of at least 100 accessions each, maintained by Italian universities, including those of Torino, Perugia, Ancona, Viterbo, Sassari, and Pavia. Although the total number of Italian common bean accessions stored *ex-situ* is remarkable, a lack of systematic investigation of these collections is evident (Piergiovanni and Lioi, 2010).

In the literature, several studies have characterized Italian common bean landraces using multidisciplinary approaches, such as Lioi et al. (2005); Marotti et al. (2007); Raggi et al. (2013); Ombra et al. (2016); Falcione et al. (2022), and (2024). Moreover, many works focus on landraces from specific Italian regions, addressing on-farm conservation and the safeguarding of traditional cultivation. In northern and central Italy, research has addressed Piemonte, Lombardia, Veneto, Trentino, Toscana, Lazio, Marche, and Abruzzo (Piergiovanni et al., 2000a and 2006; Sicard et al., 2005; Piergiovanni and Lioi, 2010; Catarcione et al., 2023), documenting morphological traits, phaseolin patterns, and population variation. In southern Italy, extensive work has been conducted in Campania, Basilicata, Calabria, Puglia, and Sicilia performing multidisciplinary characterizations, as well as investigating nutritional and culinary traits (Limongelli et al., 1995; Piergiovanni et al., 2000b, c and Piergiovanni et al., 2006; Lioi et al., 2012; Mercati et al., 2013; Scarano et al., 2014; De Luca et al., 2018; Lioi et al., 2019). Finally, Losa and colleagues in 2025 focused on bean landraces, specifically Borlotto type, from Lombardia, Emilia-Romagna and Veneto.

Comprehensively, these studies confirm the extensive genetic diversity of Italian common bean landraces and underscore that effective conservation strategies require a thorough understanding of the germplasm to prevent the loss of valuable genetic resources and to avoid preserving genetically redundant populations.

Compared with *P. vulgaris*, for which numerous characterization studies exist, considerably fewer investigations have focused on *P. coccineus* in Italy. Negri and Tosti (2002) analysed five *P. coccineus* landraces reproduced on-farm in central Italy (Umbria, Lazio, and Abruzzo), characterizing them morphologically and with molecular markers; their results demonstrated the distinct genetic identity of these landraces shaped by human and environmental selection pressures. Sicard et al. (2005) examined diversity patterns in local varieties of *P. vulgaris* and *P. coccineus* from the Marche region, where both species are traditionally cultivated together, and characterized nine *P. coccineus* local varieties collected from 11 farms. Acampora et al. (2007) investigated variation in seed traits and molecular markers across 21 runner bean landraces from eight Italian regions (Lombardia, Friuli Venezia-Giulia, Emilia-Romagna, Lazio, Abruzzo, Molise, Campania, and Calabria), showing that Italian farmers developed distinct landraces from ancestral Mesoamerican introductions through selection for seed size and colour, while occasional gene flow maintained intra-landrace variability.

A few years later, Mercati et al. (2015) analysed 20 populations from central Italian runner bean landrace “Fagiolone” alongside 41 Italian and Mesoamerican accessions. Their findings also highlighted the influence of farmers’ management practices, including seed exchange and selection, as well as past introductions of morphologically similar but genetically distant material (Mercati et al., 2015). Finally, Giupponi et al. (2018) focused on the agronomic characterization of the “Copafam” landrace from the Brescia pre-Alps in the Lombardia region.

These studies identify various Italian regions where the cultivation of bean landraces, both common and runner bean, is well established; however, only two works have specifically explored the genetic diversity of runner bean in Lombardia. Nevertheless, in this specific region of Italy, there are several collections of landraces of both *P. vulgaris* and *P. coccineus*, which are preserved at specialized centres, such as the Germplasm Bank of the Università di Pavia, the CREA Research Centre for Genomics and Bioinformatics in Montanaso Lombardo, and the DI.PRO.VE.S. at Università Cattolica del Sacro Cuore in Piacenza (Losa et al., 2025). These institutes not only conserve various accessions of local varieties but also maintain these materials for self-consumption purposes, operated by agricultural enterprises and the interest of private individuals in traditional local varieties.

Since common bean and runner bean landraces are more beneficial to the environment and biodiversity than conventional systems, since they could be used as a genetic resource against environmental stresses caused by a multitude of biotic and abiotic factors (Caproni et al., 2018), and since they can be also used to improve the quality of currently cultivated varieties (Márquez et al., 2024; Holsinger and Weir, 2009), this study aimed to recover, conserve, and characterize both morphologically and genetically a pool of landraces of common bean (*Phaseolus vulgaris* L.) and runner bean (*Phaseolus coccineus* L.) present in Lombardia, which are threatened by extinction and genetic erosion.

To achieve this goal, eight accessions of common bean and seven accessions of runner bean were sampled in the Lombardia region. The selection of these landraces was based on their high local relevance, limited prior characterization, and the availability of seeds within well-defined cultivation areas. Emphasis was placed on identifying non-”borlotto” types and varieties with a strong and documented link to local history, culture, and territory, in order to capture underexplored components of regional agrobiodiversity.

Traditional landraces of *P. vulgaris* in Northern Italy share a common history of on-farm conservation through family-based seed saving, mainly documented from the mid-20th century, although some types can be traced back to the 19th century (e.g., “Sargentone” and “Aquila” groups). Maintained in geographically marginal areas, these genetic resources have undergone local adaptation, leading to the development of distinct ecotypes, often derived from older commercial varieties or introduced materials. Despite this shared origin, they exhibit considerable phenotypic and functional diversity, including snap, dry, and dual-purpose types, as well as distinctive seed and pod traits associated with local nomenclature and cultural identity. Their uses are typically stage-dependent, with immature pods consumed as fresh vegetables and mature seeds used in soups and stews, reflecting a close relationship between genetic diversity and traditional food practices (Stagnati et al., 2023). Similarly, the selected landraces of *P. coccineus* share a common historical trajectory, linked to their introduction from Mesoamerica in the late 16th century and subsequent long-term local adaptation and conservation, particularly in mountainous environments. Although cultivation is generally documented from the 19th century onward, oral histories indicate continuous transmission within families and small communities. These landraces display notable diversity in seed color, flower morphology, and growth habit, as well as differences in geographical distribution. Their strong association with subsistence agriculture is reflected in local nomenclature (e.g., terms meaning “hunger killer”), underscoring their historical importance as staple food resources. Traditional uses are largely consistent and stage-dependent: while immature seeds may be consumed fresh, they are primarily used as dry beans in soups, stews, and cereal-based dishes, highlighting their key role as a plant-based protein source in rural diets (Stagnati et al., 2023).

Once these accessions were collected, they were morphologically characterized *in-situ*, and a genetic characterization was performed using the genotyping-by-sequencing approach to investigate their genetic biodiversity and structure.

## 2 Materials and methods

### 2.1 Germplasm and field management

Field surveys conducted by personnel of Università degli Studi di Pavia and Università Cattolica del Sacro Cuore allowed the identification of the germplasm used in the present study. Eight common bean and seven runner bean traditional varieties were sampled in in hills and mountain areas in Lombardia; detailed information on the germplasm both of common and runner bean used in this study are provided in **Supplementary Table 1**. Bean accessions were cultivated *in-situ*, morphological descriptors were evaluated according to the CPVO TP/9/1 protocol.

### 2.2 Plant material and DNA extraction

Leaf samples for DNA extraction were collected from young seedlings grown in Petri dishes. Globally, 65 different individuals of common bean were considered, specifically: 5 for “Fagiolo Anellino dell’Oltrepò Pavese”, “Fagiolo Dorato della Valchiavenna”, and “Bigliolo di Ghiaie di Corana”; 6 for “Fagiolo di San Giacomo Filippo”; 10 for “Fagiolo Anellino della Valchiavenna”, and “Sargentone di Valvestino”; 12 for “Aquila dell’Oltrepò Pavese”, and “Mangiatutto Giallo dell’Oltrepò Pavese”. Regarding the runner bean, a total of 168 different individuals were considered: 4 individuals for “Copafam della Val Camonica”; 25 for “Storo della Valle del Caffaro”; 25 for “Diavoli della Valle del Caffaro”; 26 for “Coccineo della Valchiavenna”; 27 for “Coccineo della Valvestino”, 30 for “Coccineo della Valmalenco”; finally, 32 for “Diavolo dell’Oltrepò Pavese”. Differences in samples size were due to limited size and/or poor germination of the seed stocks obtained during sampling activities.

Genomic DNA was extracted with GenElute™ Plant Genomic DNA Miniprep Kit (Merck Life Science s.r.l. Darmstadt, Germany) following manufacturer instructions with minor corrections reported in Stagnati et al. (2021) consisting in the addition of 5% W/V polyvinylpyrrolidone (PVP) in the lysis step to enhance the removal of polyphenols and performing all centrifugation steps at 4 °C. The extracted DNA was then evaluated for quality and quantified using NanoPhotometer N80 (IMPLEN) and visualized on 1% agarose gel electrophoresis stained with Eurosafe nucleic acid stain (EuroClone).

### 2.3 Sequencing and bioinformatic analysis

Genotyping, raw reads processing and variant calling were performed at CD – Genomics, The Genomics Services Company (SUITE 111, 17 Ramsey Road, Shirley, NY 11967, USA) with double digest Restriction Associated DNA sequencing approach (ddRAD) using *PstI-BfaI* as restriction enzymes. Pair-end sequencing was performed on Illumina^®^HiSeq PE150 platform (Illumina, California, USA). The original sequencing data acquired by high-throughput sequencing platforms were transformed to sequence reads by base calling with the CASAVA software. Raw data obtained from sequencing were filtered for adapter contamination and reads with more that 10% of uncertain nucleotides and base quality less than 5 (Q ≤5) reads were discarded.

Using BWA-MEM v0.7.17 (Li, 2013), the resulting clean reads of the 65 common bean and 168 runner bean samples were mapped against the reference genome of *P. vulgaris* L version 1 (genome assembly PhaVulg1_0, GCA_000499845.1) (Guerra-García et al., 2017). Although a genome assembly for *Phaseolus coccineus* is currently available in NCBI (GCA_037954025.1), it remains relatively recent and not yet fully annotated. For this reason, the *P. vulgaris* reference genome, well annotated and extensively used in genomic studies, was selected for read alignment. Given the close phylogenetic relationship and high level of genomic synteny between the two species, this approach has been previously adopted and is can be considered appropriate for population genomic analyses (Guerra-García et al., 2017).

For both species, after the alignment step, duplicates were removed by SAMtools v1.10 (Danecek et al., 2021). Finally, single nucleotide polymorphisms (SNPs) were detected using SAMtools mpileup (v1.10; Danecek et al., 2021). The resulting variants were filtered for quality (QUAL > 30) and read depth (DP > 10) using BCFtools v1.10.2 (Danecek et al., 2021). To retain only high-confidence SNPs, indels and multiallelic variants. At this step the distribution of SNPs across the genome was plotted both for *P. vulgaris* and *P. coccineus* using the CMplot package in R (v.4.1.3) (Yin et al., 2021; R Core Team, 2022). For both species the respective genetic datasets were filtered for missing data (common bean > 0.9, and runner bean > 0.8); minor allele frequency (MAF) lower than 0.05; and SNPs with missing call rates higher than 0.2 (Abebe et al., 2015; Stagnati et al., 2019; Lioi et al., 2019; Caproni et al., 2023). In addition, a linkage disequilibrium (LD) pruning step was performed with the command --indep-pairwise 150 5 0.5 of the PLINK v.1.9, as recommended in the PLINK manual (Purcell et al., 2007) and in a previous study (Calus and Vandenplas, 2018).

### 2.4 Population genetics analysis

Genetic structure and diversity of common and runner bean varieties under study were elucidated through principal component analysis (PCA) (Greenacre et al., 2022; Lezzi et al., 2023) using PLINK v.1.9 (Chang et al., 2015) for principal components calculation. R software v. 4.1.3. was used for visualizing the resulting plot. Analysis of Molecular Variance (AMOVA) was performed to quantify the partitioning of genetic variation within and among groups using the poppr.amova function implemented in the R package Poppr (Kamvar et al., 2014, 2015; R Core Team, 2022). Genetic differentiation among populations was assessed using the Phi statistic (PhiPT). The significance of the variance components and PhiPT values was evaluated through 1,000 permutation tests, following the approach described by Andrijanić et al. (2023).

Population structure was investigated using the ADMIXTURE v.1.3.0 package (Alexander et al., 2020), with the number of clusters (K) ranging from 2 to 15. To assess the quality of the clustering and infer the most likely K-value, we estimated the cross-validation error for each K-value. The results of the ADMIXTURE analysis were visualized using the *Pophelper* R package (Francis, 2017).

To visualize genetic relationships among accessions, hierarchical clustering was performed using the UPGMA algorithm based on SNP-derived genetic distances. The resulting dendrogram was generated in R using the package ape (Paradis and Schliep, 2019), and branch support was evaluated with 1000 bootstrap replicates.

Observed and expected heterozygosity, inbreeding coefficient (F), and population pairwise fixation index (*F_ST_*, Weir and Cockerham, 1984) were computed with VCFtools (Danecek et al., 2011) using the “--het” and “--weir-fst-pop” functions, respectively for both species. Graphs were subsequently plotted with *ggplot2* and *heatmap.2* packages in R (Wickham, 2014; R Core Team, 2023; 196 Warnes et al., 2024).

To investigate local adaptation of the landraces understudy genome scans for outlier loci were conducted using the *pcadapt* package on R (Privé et al., 2020; R Core Team, 2023). First, to determine the appropriate number of principal components (K), multiple models (K = 2–20) were tested. Model fit was evaluated using the Genomic Inflation Factor (GIF) and scree plot inspection. Genome-wide association statistics were then computed, and z-scores, chi-square statistics, and p-values were produced for each SNP. Multiple testing correction was applied using the Benjamini–Hochberg False Discovery Rate (FDR) with a significance threshold of α = 0.001 (Privé et al., 2020; R Core Team, 2023). Finally, SNPs were mapped to reference chromosomes, ordered by genomic position, and visualized using Manhattan plots (Privé et al., 2020; R Core Team, 2023).

Moreover, Isolation by Distance (IBD) was assessed using a Mantel test with Pearson’s correlation and 9,999 permutations, as implemented in the *vegan* package (Oksanen et al., 2026; R Core Team, 2023). Additionally, linear regression was used to quantify the relationship between genetic and geographic distances.

## 3 Results

### 3.1 Phaseolus vulgaris

#### 3.1.1 Morphological characterization of *Phaseolus vulgaris* L. germplasm

In the present study eight different accessions of *P. vulgaris* L. have been considered.

“Fagiolo Anellino dell’Oltrepò Pavese” is a type of bean where the entire pod is consumed, known as “snap bean”, with a climbing growth habit (**Supplementary Table 1**; **Supplementary Figure 1**). The pod is stringless, strongly curved and, at maturity, is uniformly green without spots. The beans are 10–12 mm long, ellipsoidal, black, shiny, with a white hilum.

“Fagiolo Anellino della Valchiavenna” is a snap bean with a climbing growth habit, flowers have purple standard and wing petals (**Supplementary Table 1**; **Supplementary Figure 1**). The pod is 15–20 cm long, 1 cm wide, and ranges from slightly to strongly curved, especially at the apical half; the apex almost always has a beak that is hooked and pressed against the ventral margin. At maturity, the surface is green with scattered to dense purple spots; when dry, it becomes wrinkled and straw-yellow with less noticeable spots. The pod is stringless. The beans, 7–8 per pod, are 15 mm long, elliptical-kidney-shaped, elongated; the surface is slightly shiny, beige-pink, with dark purple or black rhomboidal spots that tend to merge into streaks, with a white hilum surrounded by orange.

“Fagiolo Dorato di Valchiavenna e Valtellina”, here after mentioned as “Dorato”, is a dual-purpose landrace, both snap bean and dry bean, with a climbing growth habit (**Supplementary Table 1**; **Supplementary Figure 1**). The pod is 13–16 cm long and 1.8-2.2 cm wide, with an elliptical cross-section, ranging from straight to slightly curved towards the tip; a nearly straight beak is centrally positioned at the apex. At maturity, the surface has slight constrictions between the seeds and varies in colour from light green to yellow, without spots; when dried, it becomes intensely yellow and wrinkled. The string is absent in young pods but present in more mature ones. The beans, 6–8 per pod, are 14–16 mm long, with shapes ranging from elliptical to almost rectangular, slightly shiny, beige-pink, and typically bear two characteristic concentric streaks (accompanied by a few rhomboidal spots) ranging in colour from light brown to gold, which darken with age; the hilum is white and surrounded by yellow-orange.

“Sargentone di Valvestino”, here referred as “Magasa”, has a climbing growth habit, with cream-white flowers, and immature pods that are green, developing reddish streaks as they mature (**Supplementary Table 1**; **Supplementary Figure 1**). The beans, exclusively for shell beans, are approximately 20 mm long, with shapes ranging from elliptical to kidney-shaped. The base colour is white on half of the seed with sparse violet spots, while the secondary colour is a wine-red covering the remaining half of the seed, including the hilum. In the violet half, there are variably shaped and sized light brown patches. The cultivation cycle lasts about 5–6 months, with harvesting around September.

“Fagiolo di San Giacomo Filippo” is a dual-purpose kind of bean with a climbing growth habit and flowers with violet corolla (**Supplementary Table 1**; **Supplementary Figure 1**). The pod is 10–11 cm long and 1.2-1.5 cm wide, flattened and straight; at the tip, there is a hook-like beak pressed against the ventral margin of the pod. The pod surface at maturity shows no constrictions between the seeds, is yellow-green with scattered to dense purple spots; when dry, it becomes wrinkled, ranging in colour from straw-yellow to light brown, with scattered or absent spots. The beans are 6–7 per pod, 9–12 mm long, with shapes ranging from rectangular to elliptical to almost round, shiny and beige-pink in colour, with dark purple-black rhomboidal spots that are scattered or coalesce to form concentric streaks or nearly cover the entire bean surface, making it black with minute brown or purple specks; the hilum is white and surrounded by orange.

“Fagiolo Aquila dell’Oltrepò Pavese” is a landrace for dry beans characterized by climbing growth habit and white flowers (**Supplementary Table 1**; **Supplementary Figure 1**). The pod is 9 cm long and 1 cm wide, straight, and stringless; the beak at the tip is central and ranges from straight to slightly curved. When dry, the pod has a smooth surface, cream-yellow in colour, with slight constrictions between the seeds; it also tends to curl helically. The beans, 7 per pod, are 11–12 mm long, nearly rectangular in shape, with a shiny and uniformly cream-white surface, except around the hilum, which is surrounded by a characteristic red-brick-colored design resembling a double-headed eagle (aquila), which gives the name to the accession.

“Fagiolo Mangiatutto Giallo dell’Oltrepò Pavese” is a climbing plant that produces flowers of a characteristic violet colour (**Supplementary Table 1**; **Supplementary Figure 1**). The pods, which are rather long, are green when young and during their development, turning yellow as they mature. The seeds are generally large, white when immature, and dark blue-black when fully mature and dry.

“Fagiolo Bigliolo di Ghiaie di Corana” is a dry bean landrace, with a climbing growth habit, and white flowers with pinkish hues (**Supplementary Table 1**; **Supplementary Figure 1**). The beans are rather elongated with an elliptical shape. The surface is shiny, half white with occasional violet speckles, and the other half (ventral side with the hilum) ranging from light to dark beige with scattered dark purple rhomboidal spots (sometimes the purple nearly covers the beige background).

#### 3.1.2 SNPs analysis of *Phaseolus vulgaris* L. germplasm

GBS sequencing of *P. vulgaris* L. yielded 302,083 variants, evenly distributed across all bean chromosomes, with few low SNP-density areas (**Supplementary Figure 2**). Raw data were inspected for missing data presence at individual level, which was very high (about 70%). The high proportion of missing data observed in the raw dataset is consistent with expectations for GBS approaches, where reduced-representation sequencing and uneven locus coverage across samples commonly result in substantial missingness (Wang et al., 2020; Munyengwa et al., 2021). Notably, missing data were broadly distributed across individuals rather than concentrated in a subset of samples, indicating that missingness primarily reflects methodological constraints rather than sample-specific quality issues.

**Figure 1:**
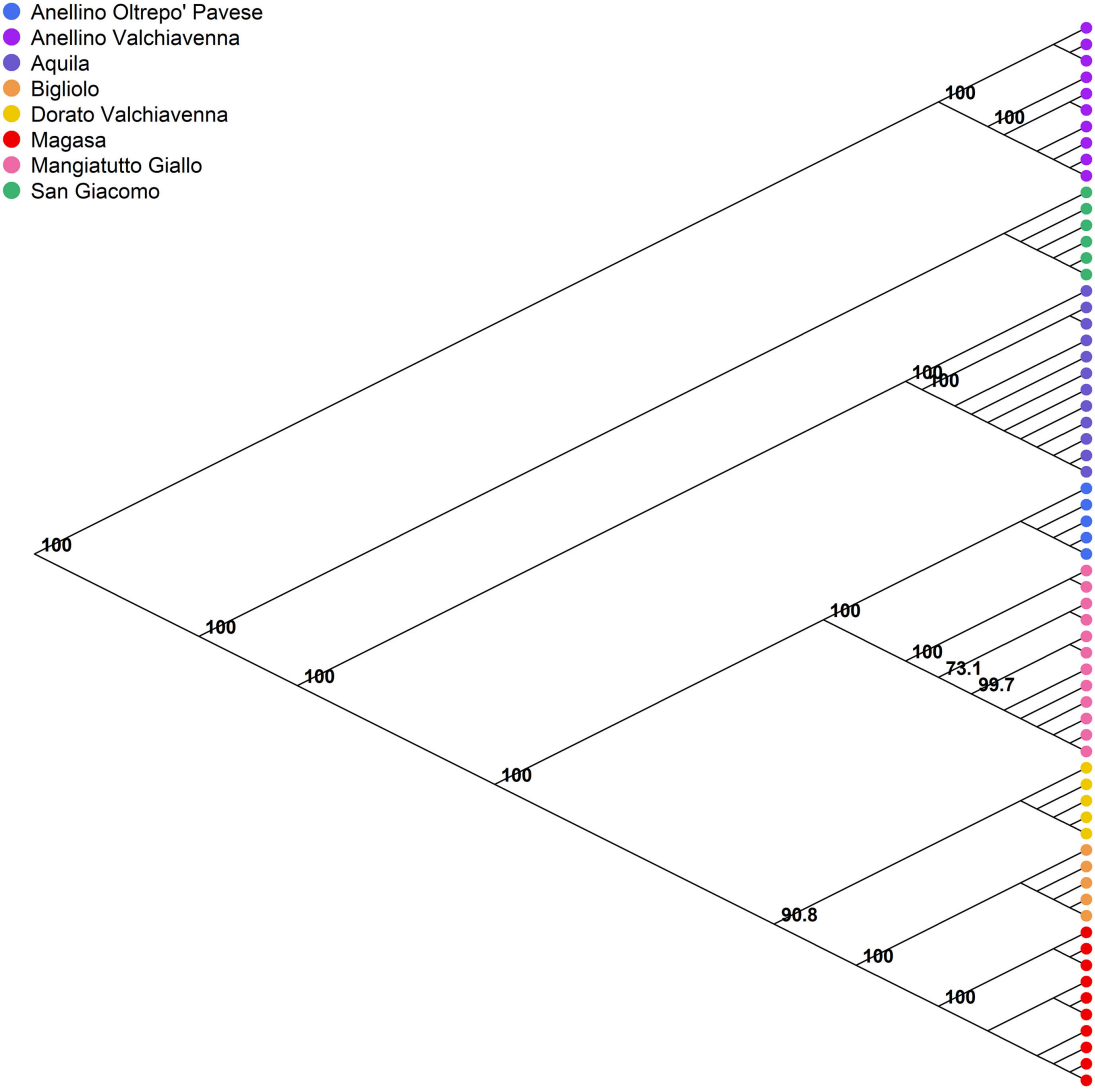
UPGMA dendrogram of 65 *Phaseolus vulgaris* L. individuals from eight landraces based on 16,622 SNP markers. Coloured symbols indicate landrace assignment; bootstrap support values are shown for major clusters.

Raw data were initially filtered for MAF and for missing data at site level resulting in 16,622 SNPs used for subsequent analysis; this SNP number is similar to those obtained by other authors (Losa et al., 2025). The final SNP dataset was used to derive a dendrogram of the common bean (**Figure 1**), which presents a very clear and defined situation. All the studied landraces form well-defined groups that include all the individuals analysed within the same accession, allowing for clear separation and highlighting existing relationships that cannot be derived from simple phenotypic observation or from the area of origin of the landraces. The two most distantly related bean populations are “Anellino di Valchiavenna” and “Fagiolo di San Giacomo Filippo”, both from the same geographical area corresponding to Valchiavenna. The third population separating from the others is the “Fagiolo Aquila”, after which the tree bifurcates. On the first branch, the “Fagiolo Dorato di Valchiavenna” is followed by the two bicolour beans: “Magasa” and “Bigliolo delle Ghiaie di Corana”, both characterized by the same colour distribution, more intense in “Magasa” and paler in “Bigliolo”. The other branch leads to two beans from Oltrepò: “Mangiatutto giallo” and “Anellino”. It is interesting to note the homonymy between the two beans “Anellino di Valchiavenna” and “Anellino dell’Oltrepò”, which are genetically distinct and distant.

**Figure 2:**
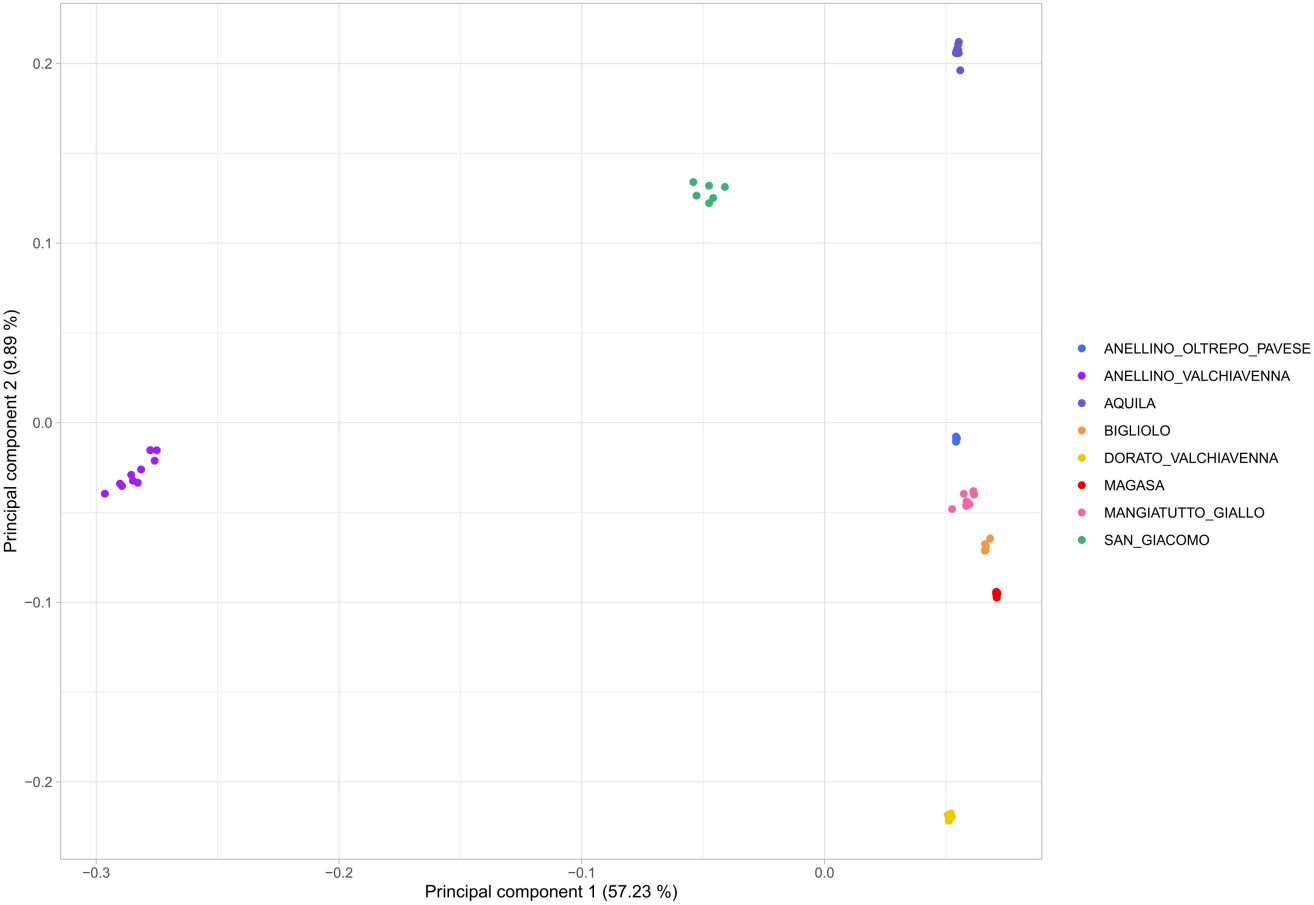
principal component analysis of 65 samples of *Phaseolus vulgaris* L. landraces from Italy; the 16,622 SNPs set was used; component 1 vs. component 2.

The Principal Component Analysis (PCA; **Figure 2**; **Supplementary Figure 3**) explains a total variability of 75.43%, with the first three principal components accounting for 57.23%, 9.89% and 8.31%, respectively. Landraces are well defined with individuals clustering strictly together. AMOVA results were consistent with the PCA analysis and revealed a strong and highly significant genetic differentiation among groups (p = 0.001). Most of the genetic variation was distributed among groups (93%), indicating that the eight identified genetic clusters were largely distinct from one another. In contrast, only 7% of the total genetic variation was found within groups, suggesting a high degree of genetic homogeneity among individuals belonging to the same cluster. Losa et al. (2025) analysing landraces ascribed at the Borlotto type, found within landrace genetic variability that caused samples belonging to the same accession to be dispersed in the dendrogram. In the present study, distinctive morphology and geographic isolation between landraces may have helped in maintaining genetic distinctiveness (Losa et al., 2025).

**Figure 3:**
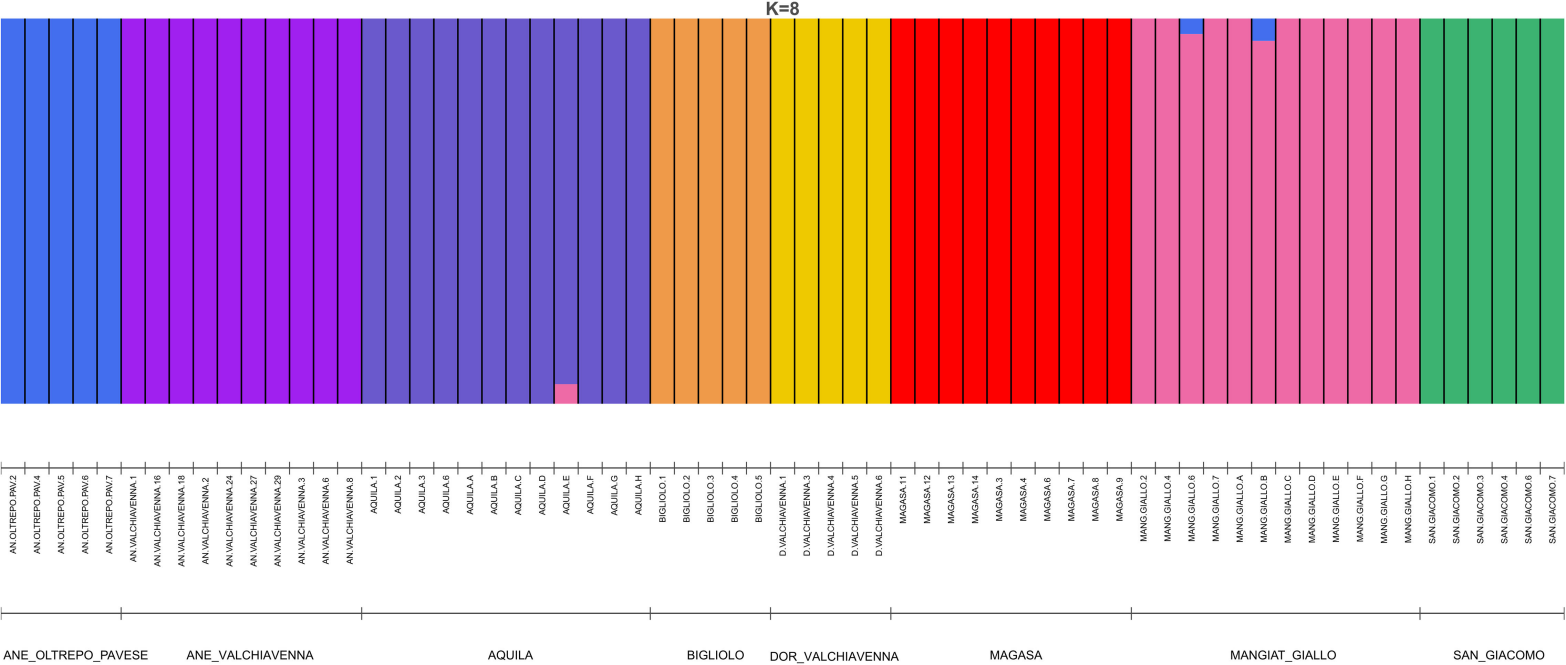
Population genetic structure at K = 8 of the 65 individuals of 8 *Phaseolus vulgaris* L. accessions evaluated in the present study. Different colours correspond to different ancestral populations.

Population structure analysis (**Figure 3**) identified eight ancestral populations that contributed to the present collection (K = 8, Cross validation error = 0.15801). This analysis clearly identifies that each studied accession originated independently, confirming findings from previous analyses. The first landrace that separates at K = 2 (**Supplementary Figure 4A**) is “Anellino della Valchiavenna” followed by “Mangiatutto Giallo” and “Anellino dell’Oltrepò Pavese” at K = 3 (**Supplementary Figure 4B**). Further, at K = 4 (**Supplementary Figure 4C**) there is the separation of “Dorato” from “Magasa” and “Bigliolo”, at K = 6 “Mangiatutto Giallo” and “Anellino dell’Oltrepò Pavese” are set apart one another while “Dorato” is rejoined to “Magasa” and “Bigliolo” (**Supplementary Figures 4C, D**); this latter is separated at K = 7 (**Supplementary Figure 4E**). From this analysis, it emerges that each variety has a proper distinctiveness and admixture analysis is consistent with the UPGMA dendrogram analysis.

**Figure 4:**
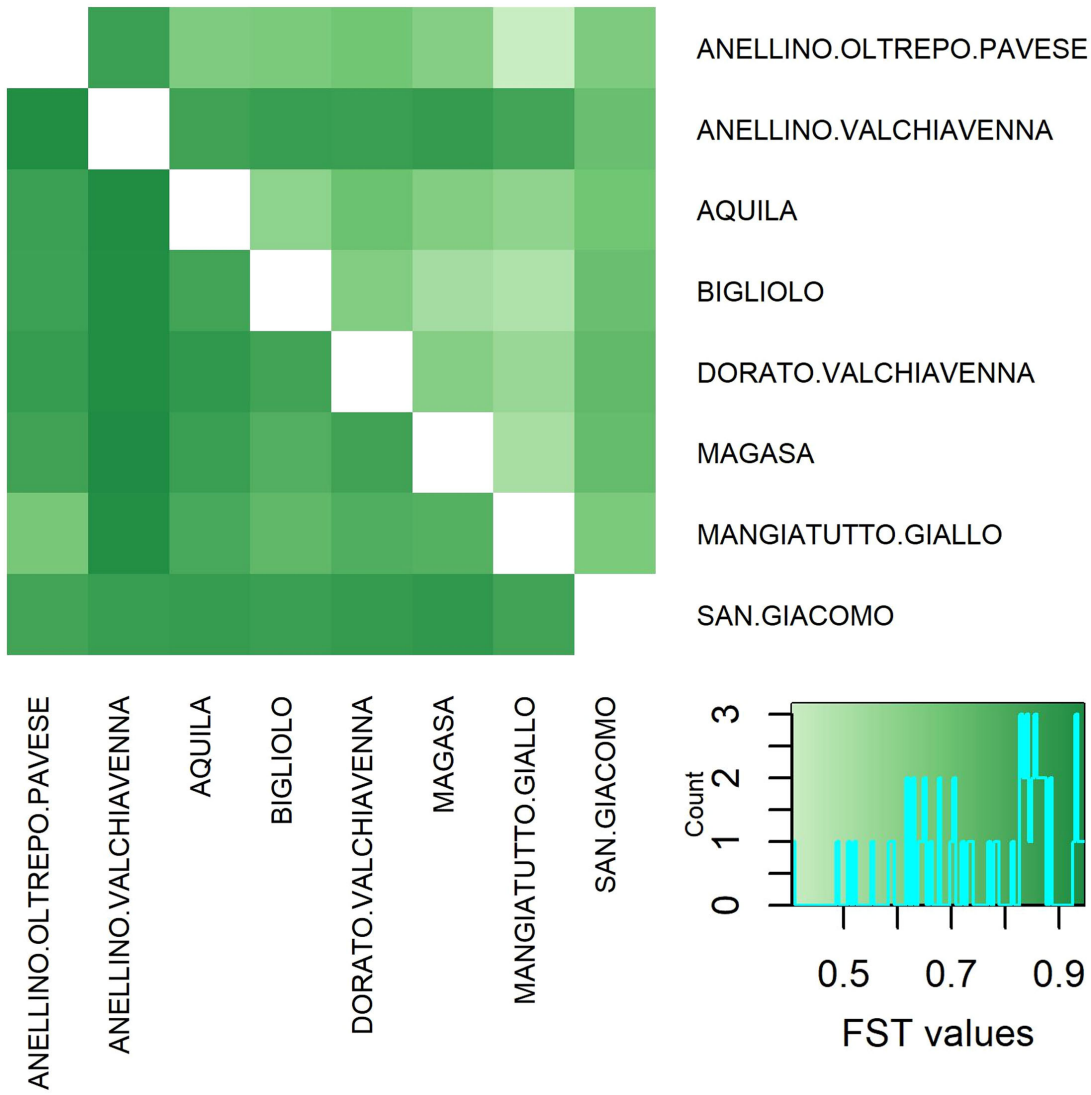
Heatmap showing genetic differentiation based on FST values of *P. vulgaris* L. samples. Above the diagonal is reported the Weir and Cockerham mean FST estimate, whereas below the diagonal is the Weir and Cockerham weighted FST estimate.

Inbreeding (F) coefficient values were very high between 0.8 and 0.95 (**Supplementary Figure 5**) consistently to the autogamous nature of common bean. Similarly, significant differences exist among expected (He) and observed heterozygosity (Ho) (**Supplementary Figure 6**), with He around 0.3 and Ho from 0.02 (“Anellino Oltrepò Pavese”, “Dorato”, “Bigliolo” and “Magasa”) to 0.05 (“Anellino Valchiavenna” and “San Giacomo Filippo”). Finally, genetic differentiation was investigated by pairwise *F_ST_* comparison (**Figure 4**) revealing a very strong genetic differentiation among common bean landraces with weighted *F_ST_* estimates ranging from 0.66, between “Anellino dell’Oltrepò Pavese” and “Magasa”, to 0.93, between “Anellino dell’Oltrepò Pavese” and “Anellino della Valchiavenna”.

For local adaptation testing PCA-based analysis revealed clear population structure, and K = 8 was selected as the most appropriate model, consistent with known biological grouping. The selected model showed a genomic inflation factor of 1.52, indicating moderate residual structure but acceptable for genome scans. After FDR correction (α = 0.001), a total of: 2,442 SNPs significantly associated with population structure were identified, representing regions potentially involved in local adaptation or demographic divergence. These SNPs were distributed across multiple chromosomes, with several regions showing clusters of highly significant signals in the Manhattan plot (**Supplementary Figure 7**), suggesting potential genomic regions under selection or strong population differentiation. A significant positive correlation between genetic and geographic distances was detected with test (r = 0.3897, p = 0.0001), indicating a moderate IBD pattern. Linear regression confirmed this relationship (R² = 0.152, p < 2.2 × 10 ¹^6^), suggesting that geographic distance explains ~15% of the observed genetic variation.

### 3.2 Phaseolus coccineus

#### 3.2.1 Morphological characterization of *Phaseolus coccineus* L. germplasm

In this study, seven different accessions of *P. coccineus* L., commonly known as “runner bean” or “scarlet runner bean,” were sampled (**Supplementary Figure 1B**) and genetically analysed. Five of these accessions were also morphologically characterized, namely: “Copafam della Val Camonica”, “Coccineo della Valchiavenna”, “Coccineo della Valvestino”, “Coccineo della Valmalenco”, and “Diavolo dell’Oltrepò Pavese”.

The remaining two accessions, “Storo della Valle del Caffaro” and “Diavoli della Valle del Caffaro”, were included in subsequent analyses because they originate from an area located at the border with Lombardia, which has historical ties to the Valvestino region.

Common feature is plant habitus, which is climbing and vigorous with plants exceeding 3 meters in height. Flowers are organized in rich racemes; corollas can be red, white or bicolour. In our collection, flowers are predominantly red in “Fagiolo della Valvestino”, “Diavolo dell’Oltrepò”, “Fagiolo Coccineo della Valchiavenna” and “Copafam”, additionally some plants can show either white or bicolour coloration. Interestingly, “Fagiolo della Valmalenco” is the only landrace characterized by the predominant presence of bicolored flowers while red and white are less abundant.

Fruits are legumes containing a small number, usually 3 or 4, large seeds. Usually, within population there is variability in seed pigmentation even in the presence of typical seed colour patterns. In “Diavolo dell’Oltrepò” the primary colour ranges from white to deep violet or beige-reddish while the secondary colour, when present, ranges from dark brown to purple or black and is distributed in small scattered rhomboidal spots or forms a single marbled patch around the hilum (marbled seed), which is white. In “Fagiolo Coccineo della Valvestino” the primary seed colour varies, generally ranging from reddish-purple to violet, sometimes white, light brown, or nearly black. The secondary colour, if present, can be brown or black, appearing in patches or marbled on the seed surface. In this bean variety, seeds with a violet primary colour are predominant, while the secondary colour can appear both in patches and marbled. Local growers prefer seeds with primary colour from reddish to purple with abundant marbling.

In “Fagiolo Coccineo della Valchiavenna”, “Fagiolo Copafam della Val Camonica”, “Fagiolo Coccineo della Valmalenco” seed pigmentation shows high intra-population variability with seeds having white, beige, light brown, pinkish, or violet as primary colour. The secondary colour is absent in beans with a primary white colour, whereas in other cases, it is always present and can be brown or black, distributed variably in sparse patches or marbled patterns.

#### 3.2.2 SNPs analysis of *Phaseolus coccineus* L. germplasm

The number of biallelic SNPs returned after sequencing the 168 individuals from different accessions of *P. coccineus* L. is 754,519; then scaffolds were removed and only 749,266 high-confidence biallelic SNPs were retained. These SNPs were plotted to analyse SNPs density showing a good distribution across all common bean chromosomes, with very little uncovered regions and few low SNP-density areas (**Supplementary Figure 8**).

The missingness at individual level was lower than 1,5% in most samples, and therefore, no filtering was performed for missing data at either the site or individual level. Instead, filtering was carried out based on MAF and missingness at site level reducing the total number of SNPs to 63,591. From this smaller dataset, further filtering was done for linkage disequilibrium with an *r^2^* of 0.5. Following these filtering steps, the final number of SNPs obtained was 28,006, which were then used for subsequent analyses.

**Figure 5:**
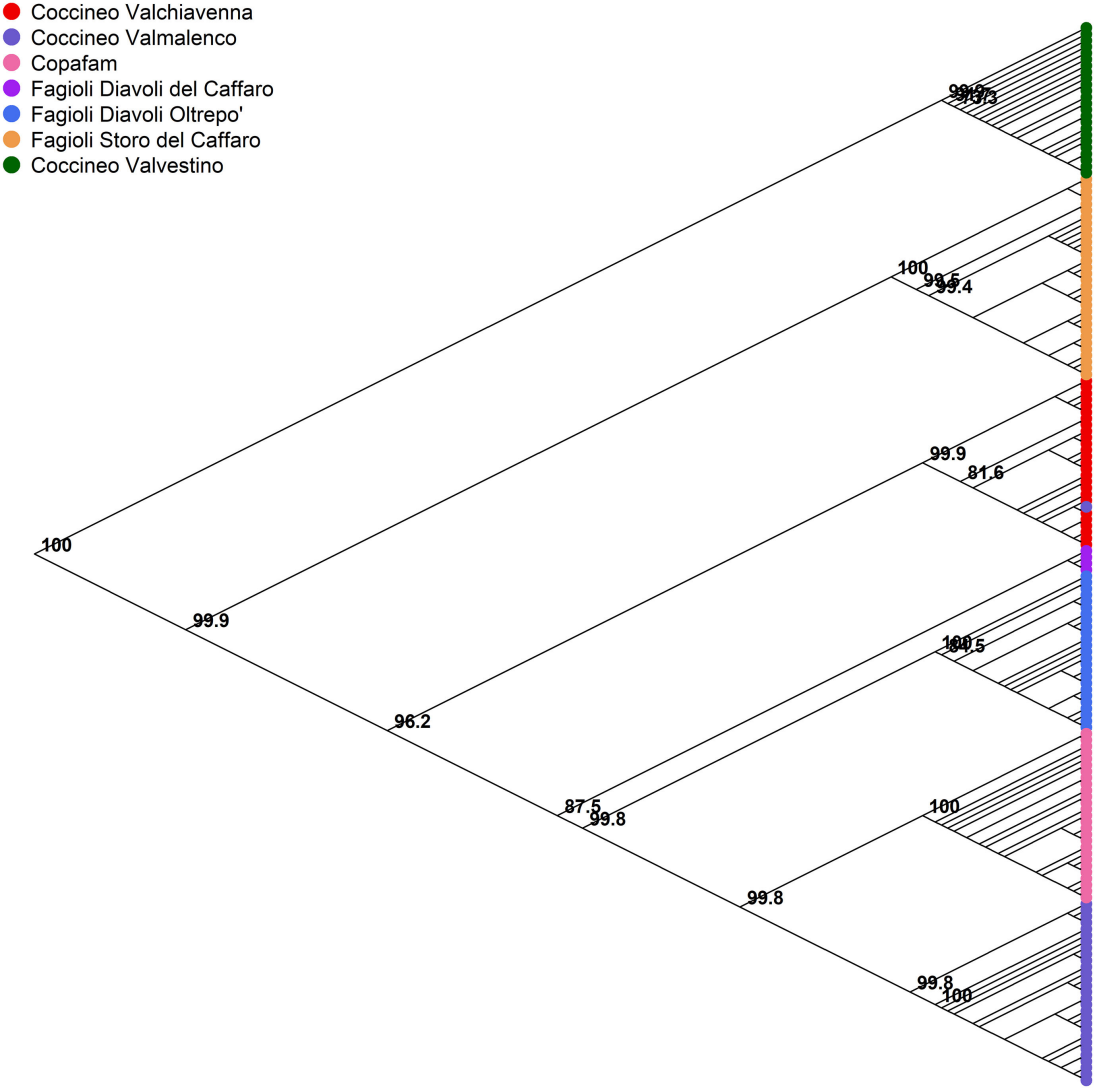
UPGMA dendrogram of 168 *Phaseolus coccineus* L. individuals from eight landraces based on 28,006 SNP markers. Coloured symbols indicate landrace assignment; bootstrap support values are shown for major clusters.

The UPGMA dendrogram constructed from all 168 individuals belonging to the different landraces (**Figure 5**) showed clear clustering of individuals within the same population, with the most divergent landrace being the outgroup “Storo del Caffaro”. After “Storo del Caffaro”, the next landrace to separate was “Diavolo Oltrepò”, followed by “Coccineo della Valchiavenna”, “Copafam”, and “Diavoli del Caffaro”, the latter collected in a location very close to that of “Storo del Caffaro”. The final branching distinguished “Valvestino” (geographically close to Storo and Ponte Caffaro) from “Coccineo Valmalenco”, which is geographically closer to Valchiavenna than to Valvestino. An off-type individual was identified within “Coccineo Valmalenco”, clustering with “Coccineo Valchiavenna”, possibly due to sample misidentification.

**Figure 6:**
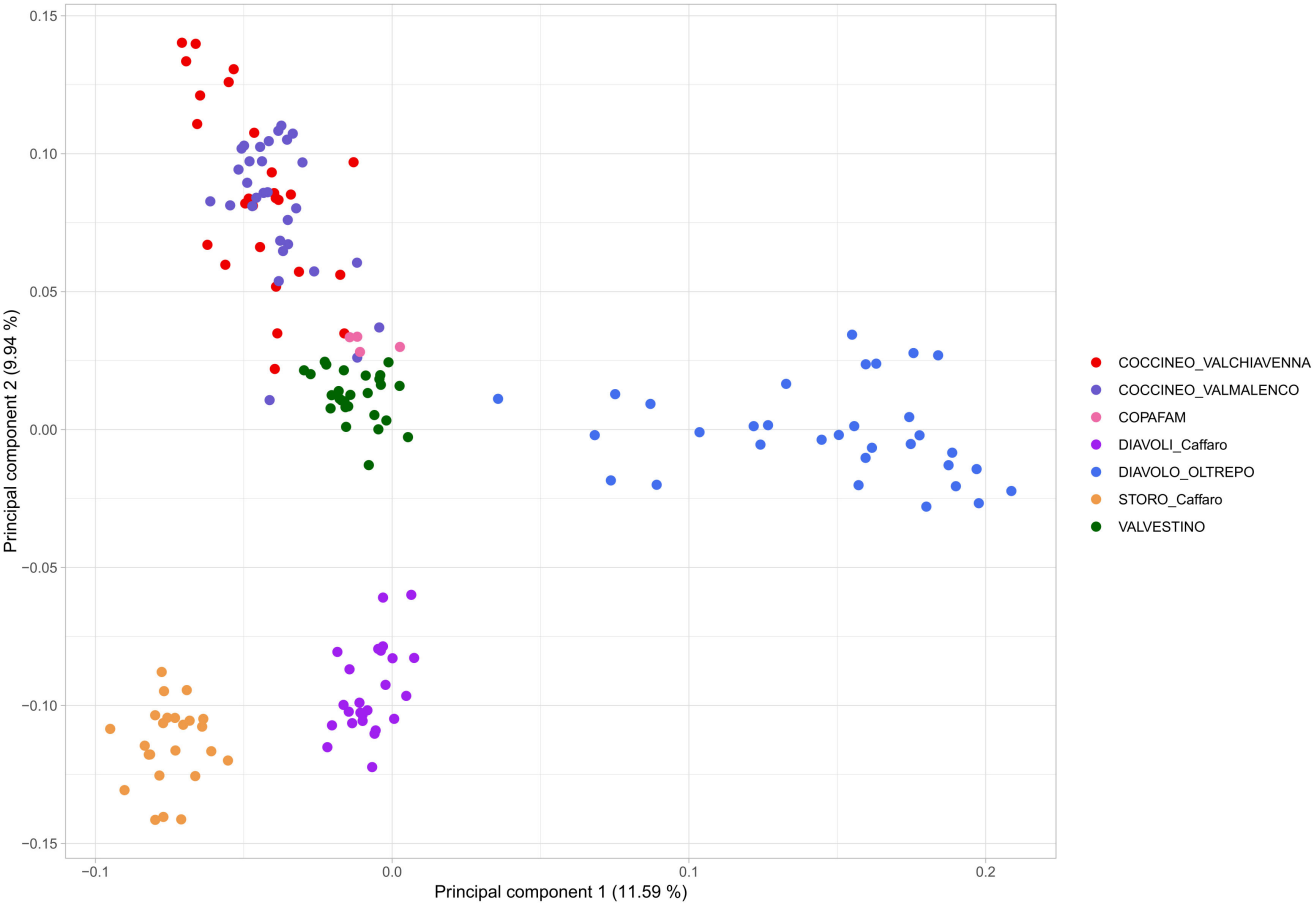
Principal component analysis of 168 samples of *Phaseolus coccineus* L. landraces from Italy; the 28,006 set was used; component 1 vs. component 2.

The Principal Component Analysis (PCA) explains a total variability of 29.98%, with the first three principal components accounting for 11.59%, 9.94% and 8.45%, respectively (**Figure 6**; **Supplementary Figure 9**). PC1 and PC2 differentiate “Diavolo Oltrepò”, “Diavoli Caffaro” and “Storo Caffaro”; “Valvestino” is distinguishable if PC3 is considered while “Coccineo Valchiavenna”, “Coccineo Valmalenco” and “Copafam” are more clustered together. Consistently with the PCA analysis, AMOVA results revealed a statistically significant population structure (p = 0.001). However, the magnitude of genetic differentiation was moderate (PhiPT = 0.12), with 12% of the total genetic variation attributed to differences among populations and 88% occurring within populations. These results indicate that, although most genetic diversity is maintained within populations, a non-random and significant genetic structure exists among populations.

**Figure 7:**
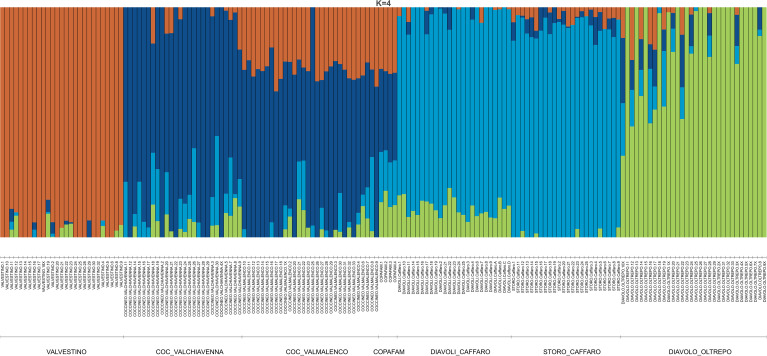
Population genetic structure at K = 4 of the 168 individuals of 7 *Phaseolus coccineus* L. accessions evaluated in the present study. Different colors correspond to different ancestral populations.

Finally, the admixture analysis conducted on the scarlet runner bean samples from Lombardia (**Figure 7**) showed that the most appropriate number of ancestral populations for these accessions is four (cross-validation error = 0.45396). All individuals displayed some degree of admixture, although in several cases the admixture proportions were low. At K = 4, most populations were well differentiated, with the exception of individuals belonging to “Coccineo Valchiavenna”, “Coccineo Valmalenco”, and “Copafam”, which exhibited similar proportions of the same ancestral populations. The only notable difference is that “Coccineo Valmalenco” and “Copafam” appear to share ancestral components with “Coccineo Valvestino”. This pattern is consistent with the PCA results, where these four local varieties cluster together. Samples from the “Coccineo Valvestino” landrace showed the lowest admixture levels, with all individuals belonging to the same cluster.

Individuals from “Diavoli del Caffaro” and “Storo del Caffaro” were genetically very similar, as most of their genetic ancestry derived from the same ancestral cluster even if “Diavoli del Caffaro” appeared to contain genetic material associated with “Diavolo Oltrepò”, an admixed population distinguishable from the other landraces. In the PCA, these three varieties, “Diavoli del Caffaro”, “Storo del Caffaro”, and “Diavolo Oltrepò”, which also share similar names and genetic material, grouped closely together yet remained clearly distinguishable. Both analyses therefore suggest a possible common origin, followed by divergence into three distinct varieties.

At K = 5 (CV error = 0.45456; **Supplementary Figure 10A**) and K = 6, (CV error = 0.45562; **Supplementary Figure 10B**), “Coccineo Valvestino”, “Storo Caffaro”, “Diavoli Oltrepò”, “Diavoli Caffaro” are clearly differentiated, while “Coccineo Valchiavenna” began to differentiate from “Coccineo Valmalenco” and “Copafam” at K = 6.

Inbreeding (F) coefficient values were very low and sometimes negative (**Supplementary Figure 11**), which is consistent with the significant differences were observed between expected (He) and observed heterozygosity (Ho) (**Supplementary Figure 12**), with significant differences between expected (He) and observed heterozygosity (Ho), with Ho frequently exceeding He. Ho ranged from 0.27 in “Diavolo Oltrepò” to 0.38 in “Copafam”, whereas He was approximately 0.29 across all samples. “Copafam” is the highest Ho and the lowest F coefficient.

**Figure 8:**
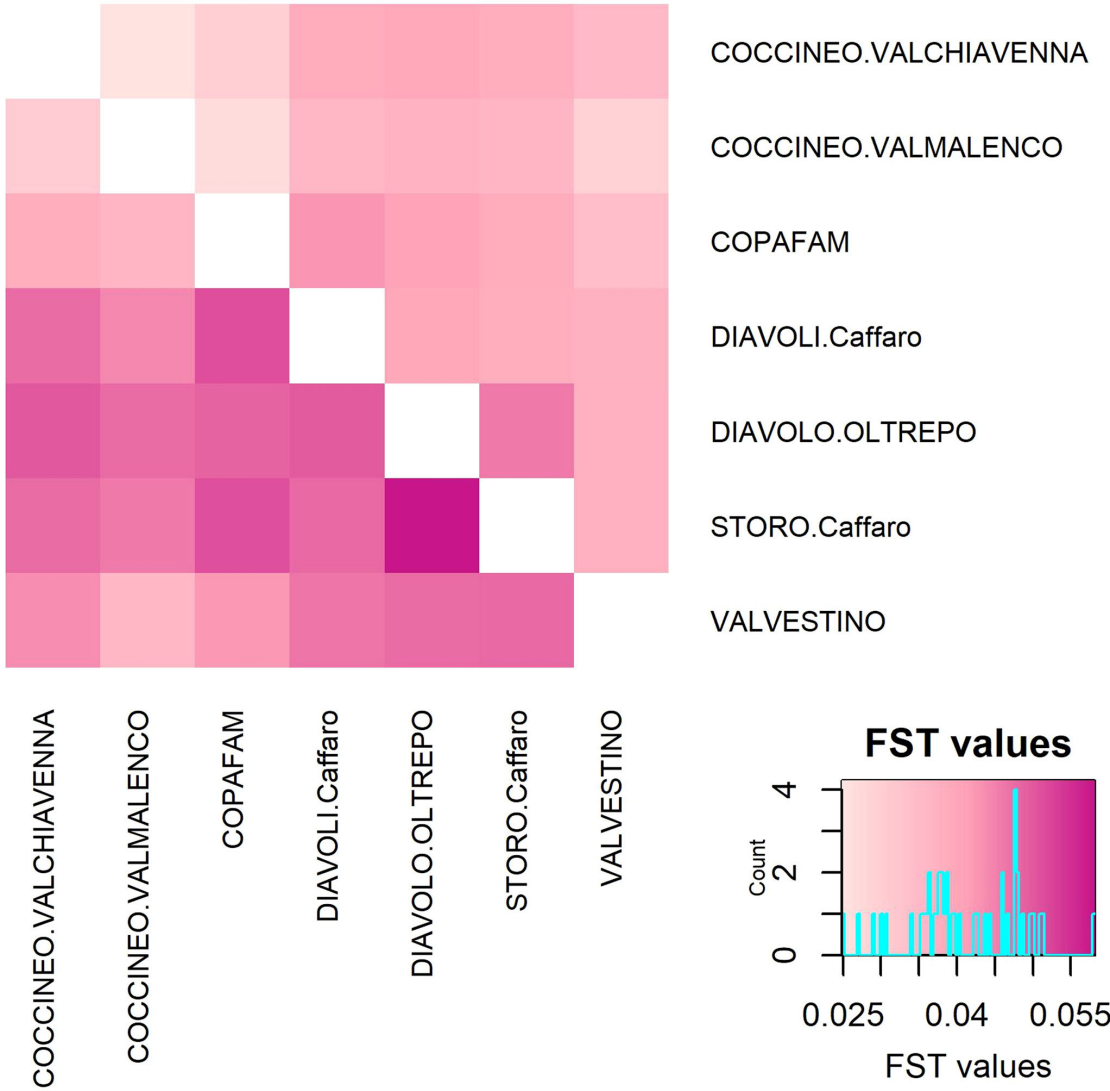
Heatmap showing genetic differentiation based on FST values of *P. coccineus* L. samples. Above the diagonal is reported the Weir and Cockerham mean FST estimate, whereas below the diagonal is the Weir and Cockerham weighted FST estimate.

In contrast to the common bean, low levels of genetic differentiation among runner bean landraces were revealed by the low *F_ST_* values (**Figure 8**), with weighted *F_ST_* estimates ranging from 0.03 between “Coccineo Valmalenco” and “Coccineo Valchiavenna” to 0.05 between “Storo del Caffaro” and “Diavolo Oltrepò”.

For local adaptation testing PCA-based genome scans identified substantial population structure, with K = 7 components used to model genetic variation. The genomic inflation factor was GIF = 1.36, indicating moderate inflation likely due to residual structure or polygenic effects. Outlier detection using FDR-corrected p-values (α = 0.001) identified 410 candidate SNPs potentially under selection. Significant SNPs were distributed across multiple chromosomes, with some clustering in specific genomic regions (**Supplementary Figure 13**). A stronger IBD pattern was observed in *P. coccineus* samples (Mantel r = 0.4639, p = 0.0001), indicating a more pronounced spatial genetic structure compared to *P. vulgaris*. Regression analysis showed a higher explanatory power (R² = 0.215, p < 2.2 × 10 ¹^6^), with ~21% of genetic variation explained by geographic distance.

## 4 Discussion

Common and runner beans (*Phaseolus vulgaris* L. and *Phaseolus coccineus* L.) are key legumes for sustainable agriculture, yet they remain under-researched and poorly improved, making them possibly vulnerable to climate change, pests, and diseases (Caproni et al., 2020; Geleta et al., 2024). In Italy, and particularly in Lombardia, a long history of cultivation has led to the development of numerous landraces, which represent an important but insufficiently explored reservoir of genetic diversity (Piergiovanni and Lioi, 2010; Raggi et al., 2019; Caproni et al., 2020). Despite the high number of accessions conserved, systematic morphological and genetic characterization, especially for runner bean, remains limited.

In the present study a comprehensive characterization of Lombardia bean germplasm was carried out combining *in-situ* morphological evaluation with GBS analysis of 8 common bean not classified as Borlotto type and 7 runner bean landraces.

### 4.1 Phaseolus vulgaris

Looking at the collection’s morphology, it can be said that is composed by landraces of climbing plant habit, which is consistent with the predominance of climbing types among Italian collections (Mauceri et al., 2025; Losa et al., 2025; Catarcione et al., 2023; Piergiovanni and Laghetti, 1999; Sicard et al., 2005). In marginal and mountain areas domestic cultivation of beans made extensive use of wooden steaks to provide support to bean plants or, especially a while ago, consociating bean with maize (Piergiovanni and Lioi, 2010; Bertolini et al., 2005).

Italy is considered a secondary centre of diversification (Losa et al., 2025), which finds support by the relevant differentiation at the level of seed size and pigmentation. In countries characterized by a high diversification of growing environments, as Italy, the process of differentiation and selection operated by farmers was more pronounced, so that each region had its own set of landraces (Lioi and Piergiovanni, 2013). The availability of improved cultivars and the industrialization of agricultural production caused the standardization of market demand towards uniform white seed (Cannellino) or well-mottled seed (Borlotto) with the disappearance of about 60% traditional varieties (Ranalli and Parisi, 2018; Losa et al., 2025), while snap bean market is dominated by varieties with green pods.

In beans, among the various organs, the most attractive is seeds. Italian landraces are highly variable in seed traits as revealed in the past by Enciclopedia agraria italiana - pubblicata sotto gli auspici della Federazione italiana dei consorzi agrari (1952-1988) and also recently by Mauceri et al. (2025) for landraces from Calabria, Catarcione et al. (2023) examining landraces from Lazio, Piergiovanni and Laghetti (1999) for landraces from Basilicata, while Venora et al. (2009) developed a system to identify landraces based on seed pictures. Even for uniform bean categories, like Borlotti, Losa et al. (2025) were able to identify several differences among landraces or cultivar, thus helping the identification process. Catarcione et al. (2023) examining seed pigmentation of 114 Italian beans found that the majority had plain colour pigmentation followed by striped types, while bicolour types are the less abundant. Similar findings have been reported also by Mauceri et al. (2025). In the present study, uniform pigmentation is the less represented (only ‘Anellino dell’Oltrepò pavese and Mangiatutto Giallo’), while ‘Aquila’ showed a pattern around the hylum and notably two landraces (‘Magasa’ and ‘Bigliolo’) are bicolour-faced. Varietal names may refer to distinctive seed-pod traits or to the area of origin (Mauceri et al., 2025) that can suggest putative genetic relatedness or synonymy. Morphology can help to a first discrimination as in the case of Anellino della Valchiavenna and Anellino dell’Oltrepò. The two landraces have the same name “Anellino” with the subsequent geographic attribution. This can suggest that they could be the same genetic entity sampled in different areas. Looking at plant morphology, it is clear that the two beans are completely different particularly at the level of pod and seeds even if varietal denomination may support, at a first glance, a case of homonymy.

Although morphological analyses highlighted valuable phenotypic differentiation among landraces, genetic characterization was necessary to evaluate their true evolutionary distinctiveness. Comparing the results of the genetic analysis together, it is possible to find consistency: in the admixture analysis the most distant varieties are “Fagiolo Anellino della Valchiavenna”, “Fagiolo di San Giacomo Filippo”, and “Fagiolo Aquila” in agreement with the dendrogram. The distribution of the other accessions is also clearly distinct, although their distribution is slightly different compared to the dendrogram. From PCA and UPGMA dendrogram analysis, it emerges that there is no clear grouping of varieties based on geographic origin, which finds support also from admixture analysis as well as from other authors (Losa et al., 2025).

The clear grouping found in the PCA and dendrogram analysis is consistent with the self-fertilizing nature of common bean and is reflected in the high values of the inbreeding (F) coefficient found in our results.

Significant differences were detected among expected and observed (Ho) heterozygosity (He), with Ho results lower. Several other works dealing with bean genetic characterization reported similar results: Ho ranged from 0 to 0.05 for common beans from Lazio (Catarcione et al., 2023), from 0 to 0.067 for common beans from Campania (De Luca et al., 2018), 0 for landraces from Croatia (Vidak et al., 2021) while for landraces sampled in Slovakia and Ukraine Ho ranged from 0 to 0.779 (Šajgalík et al., 2019) and 0.34 for Italian Borlotti (Losa et al., 2025). The present landrace collection derives from mountain and isolated areas, which may have favoured the distinctiveness of the populations (Losa et al., 2025). Negri and Tiranti (2010) reported that common bean landraces reproduced *in-situ*, retained the same level of genetic variability while *ex-situ* reproduction cause fixation because of necessity to adaptation to new environments. Genetic analysis of the present collection was carried out on original samples or on samples reproduced *ex-situ* only once but revealing a genetic condition very similar to those of stable inbred a situation compatible with the autogamous reproductive nature of common bean.

The patterns of genetic differentiation observed in *P. vulgaris* are highly consistent across multiple analytical approaches. Pairwise F_ST_ estimates revealed extremely high levels of divergence among landraces (0.66–0.93), indicating strong population structure and limited gene flow. This pattern is further supported by the significant IBD signal, with geographic distance explaining approximately 15% of the observed genetic variation. Together, these results suggest that spatial separation contributes to genetic divergence, although additional factors may have also played a role.

In this context, the large number of SNPs identified by *pcadapt* is consistent with the overall genomic pattern, since the latter method detects loci associated with population structure rather than selection alone, confirmed also by AMOVA analysis, which highlight a strong differentiation between populations. Moreover, the extensive genome-wide differentiation revealed by F_ST_ and IBD analyses naturally leads to a high number of significant associations. This effect is further amplified by the predominantly selfing mating system of common bean, which reduces effective recombination and increases linkage disequilibrium, allowing large genomic regions to diverge together.

Overall, these findings indicate that genetic differentiation in *P. vulgaris* is widespread across the genome and shaped by a combination of geographic isolation, limited gene flow, and strong population structure. Consequently, the large number of SNPs identified likely reflects both demographic processes and potential adaptive variation, rather than a small set of isolated targets of selection.

### 4.2 Phaseolus coccineus

The comparison of the morphological characterization of the two species under study shows that, whereas common bean varieties display marked variation in plant architecture, flower traits, and seed morphology, such differences are much less evident in runner bean. In runner bean, indeed, plants and seeds of different varieties are largely similar, with the main distinction resulting from local selection for white or purple seed coat colour (Sicard et al., 2005; Acampora et al., 2007; Giupponi et al., 2018). Three flower colour types (i.e. red, white, and red–white) were observed; however, these phenotypes are not supported by clear genetic differentiation. Several hypotheses may explain this pattern in runner bean, including aspects related to its reproductive biology. Runner bean is predominantly cross-pollinated, with a high rate of outcrossing, which may have facilitated ongoing genetic recombination and limited the emergence of well-defined genetic groups (Schwember et al., 2017). Additionally, the circulation of seeds between cultivation areas, in combination with insect pollination, may have promoted genetic connectivity and constrained the differentiation of distinct varieties and genetic groups (Negri and Tosti, 2002; Mercati et al., 2015).

To complement the morphological characterization, the genetic diversity and structure of the collected landraces were next examined. Results obtained in this second part of the analysis indicate a relatively low level of genetic differentiation among the investigated landraces. Although clustering analyses (dendrogram, PCA, and admixture) revealed differentiation among some landraces, particularly “Diavoli Caffaro”, “Storo Caffaro”, “Diavolo Oltrepò”, and “Coccineo Valvestino”, other varieties appeared highly similar despite their geographic separation, such as “Coccineo Valmalenco” and “Coccineo Valchiavenna”. Reduced genetic differentiation among cultivated materials compared with wild accessions is commonly reported and can be attributed to the domestication process (Negri and Tosti, 2002; Mercati et al., 2015; Schwember et al., 2017), as well as to limited introgression from wild forms (Giupponi et al., 2018).

Inbreeding (F) coefficient values were very low, consistently with the significant differences observed between expected and observed heterozygosity, with expected He lower than Ho values. Lack of heterozygosity has been reported by Spataro et al. (2011) analysing Mesoamerican and European landraces, while opposite situation is reported by Guerra-García et al. (2017). Negative F coefficients are generally associated with excesses of heterozygosity as previously reported (Guerra-García et al., 2017), thus contributing to avoid inbreeding depression, this result is consistent with the allogamous pollination of runner bean (Schwember et al., 2017). Finally, in contrast to the common bean, low levels of genetic differentiation among runner bean landraces were revealed by the low *F_ST_* values, consistent with AMOVA results, indicating weak overall genetic differentiation among landraces, suggesting substantial gene flow and limited genome-wide divergence. However, a significant IBD pattern (Mantel r = 0.4639, p = 0.0001; R² = 0.215) demonstrates that geographic distance explains a considerable proportion of genetic variation, indicating that differentiation is spatially structured rather than random. SNPs identified by *pcadapt* as significantly associated with population structure likely reflect localized genomic differentiation driven by spatially varying selective pressures. The coexistence of low overall differentiation and a strong IBD signal suggests that gene flow homogenizes much of the genome, while gradual divergence accumulates with geographic distance. This pattern is consistent with the biology of runner beans, indeed, it is important to note that this species is predominantly cross-pollinated, a feature that may have promoted continuous genetic reshuffling and hindered the formation of sharply differentiated genetic groups (Schwember et al., 2017). Regardless, the limited genetic differentiation observed has important implications for the conservation of local runner bean populations. In particular, maintaining varietal purity during cultivation, especially for seed production intended for subsequent seasons, is crucial to prevent genetic mixing through the introduction of material from other Italian regions.

## 5 Conclusions

Given the recognized value of common and runner bean landraces for environmental sustainability and biodiversity conservation, as well as their role as genetic resources for enhancing resilience to biotic and abiotic stresses and improving modern cultivars (Caproni et al., 2018; Márquez et al., 2024), this study focused on the recovery, conservation, morphological and genetic characterization of *Phaseolus vulgaris* L. and *Phaseolus coccineus* L. landraces from Lombardia region. To this end, eight common bean accessions (65 individuals) and seven runner bean accessions (168 individuals) were sampled, morphologically characterized *in-situ*, and genetically analysed using a genotyping-by-sequencing approach to assess their genetic diversity and population structure.

These landraces reflect long-term on-farm conservation and local adaptation, resulting in diverse and often highly localized ecotypes, closely linked to traditional agroecosystems, particularly in marginal and mountainous areas (Stagnati et al., 2023; Lezzi et al., 2023, 2026). Their uses are largely stage-dependent and complementary: *P. vulgaris* is consumed both as fresh pods and as dry seeds in soups and stews, whereas *P. coccineus* is primarily utilized as a dry bean in similar preparations, often combined with cereals (Stagnati et al., 2023). Overall, their enduring role in traditional diets highlights the strong connection between genetic diversity, cultural heritage, and food practices, as well as their historical importance as a key plant-based protein source in rural communities (Stagnati et al., 2023).

The results support clear separation and differentiation among landraces as a consequence of centuries of cultivation in Alpine and Apennine valleys, contrasting with patterns reported for other vegetable species of American origin (Negri and Tosti, 2002; Schwember et al., 2017; Giupponi et al., 2018). Among common bean landraces, a pronounced genetic structure was observed, with each variety showing strong genetic distinctiveness regardless of geographic proximity.

The molecular and morphological data generated in this study provide valuable resources for common bean breeding, supporting the development of improved cultivars with traits relevant for more sustainable production. Moreover, this work represents an important starting point for the genetic and morphological characterization of runner bean landraces in Italy, a species that remains comparatively understudied.

Finally, this study contributes to the development of conservation strategies for local crops and supports their valorisation in the marketplace as territory-linked, high added-value products, with particular relevance for marginal mountain economies.

Furthermore, at least for *P. vulgaris*, given the possibility of distinguishing local entities well, it can also be assumed that the study presented here can help, in addition to morphological distinctiveness, to register new entities in the context of conservation of traditional agronomic diversity, using regulatory tools at EU level (Directives for horticultural landraces) or at Italian national level (law number 194/2015).

## Data Availability

The VCF data associated with this study have been deposited in the European Variation Archive (EVA) at EMBL-EBI under project accessions PRJEB113854 (*Phaseolus vulgaris*) https://www.ebi.ac.uk/eva/?eva-study=PRJEB113854 and PRJEB114110 (*Phaseolus coccineus*) https://www.ebi.ac.uk/eva/?eva-study=PRJEB114110. The corresponding variant analyses are available under accessions ERZ29504440 and ERZ29504526, respectively.
