# Supplementary material for: Genetic diversity of common beans (Phaseolus vulgaris L.) and runner beans (Phaseolus coccineus L.): a case study from in-situ preserved landraces in Northern Italy
Source: Front Plant Sci. 2026 Jul 17;17:1838084. doi: 10.3389/fpls.2026.1838084 (PMC13423885; doi:10.3389/fpls.2026.1838084)
Supplement: Supplementary file 1 [file DataSheet1.docx]

***Frontiers in Plant Science* Supporting Information**

**Article title: Genetic diversity of Common beans (*Phaseolus vulgaris* L.) and Runner beans (*Phaseolus coccineus* L.): a case study from *in-situ* preserved landraces in Northern Italy**

**Authors:** Alessandra Lezzi^a^, Elena Petretto^a^, Alessandra Lanubile^a,b^, Francesco Ferrari^c^, Graziano Rossi^c^, Adriano Marocco^a,b^, Lorenzo Stagnati^a,b^, Matteo Busconi^a,b^

a. Università Cattolica del Sacro Cuore - Dipartimento delle Scienze delle Produzioni Vegetali Sostenibili, DI.PRO.VE.S

b. Centro di Ricerca sulla Biodiversità e sul DNA antico (BioDNA), Università Cattolica del Sacro Cuore

c. Università degli Studi di Pavia - Dipartimento di Scienze della Terra e dell’Ambiente, DSTA

Corresponding Author: Lorenzo Stagnati

Email: [lorenzo.stagnati@unicatt.it](mailto:lorenzo.stagnati@unicatt.it)

The following Supporting Information is available for this article:

**Table S1:** Accession name and attitude, sampling location, collection year and conservation site of the germplasm used in this study.

**
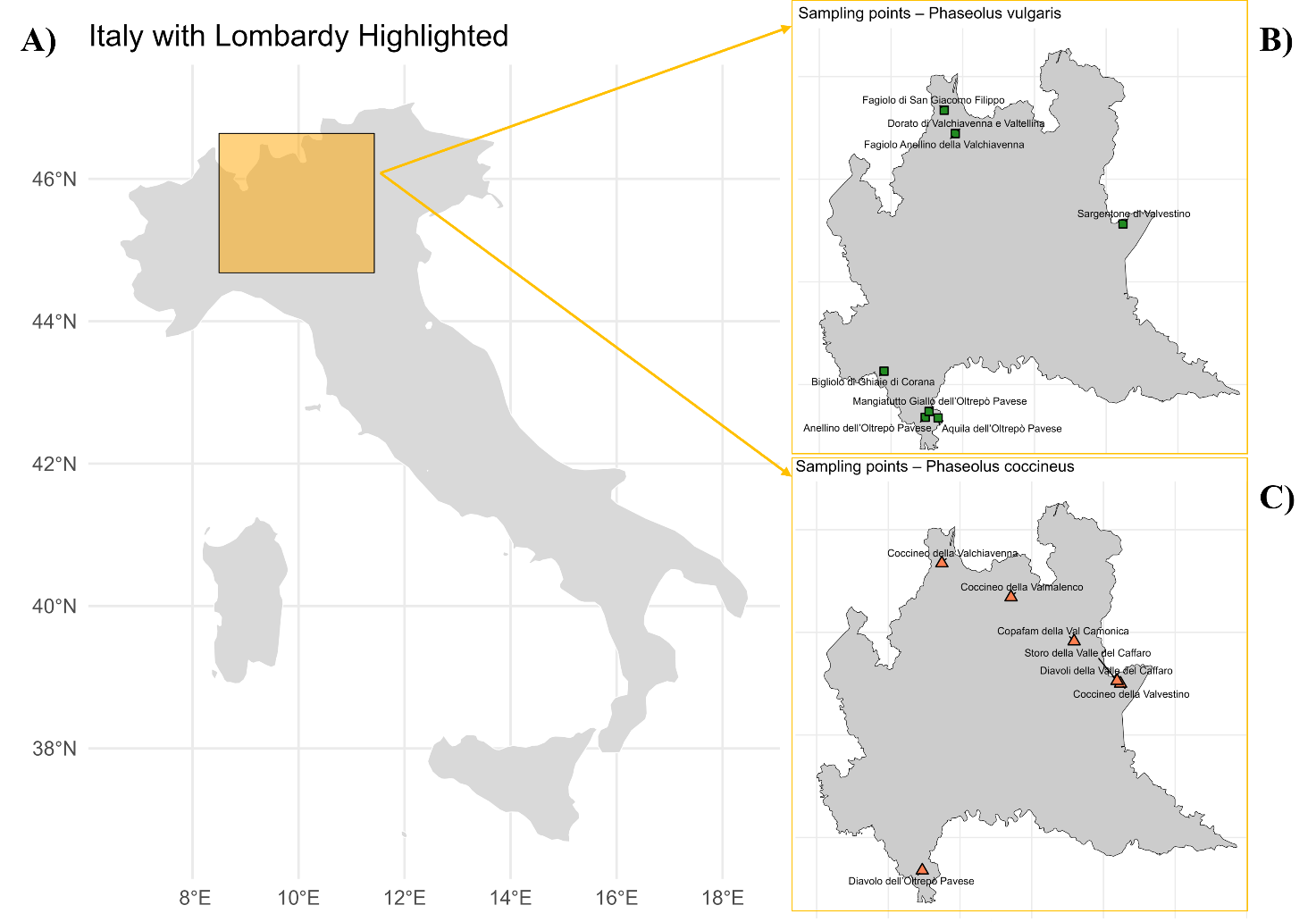
**

**Figure S1**: map of the collection sites of the two species considered in this study. A) Map of Italy with the Lombardy region highlighted in orange; B) sampling points of the 8 *Phaseolus vulgaris* samples, indicated by green squares; C) sampling points of the 7 *Phaseolus coccineus* samples, indicated by salmon-colored triangles.


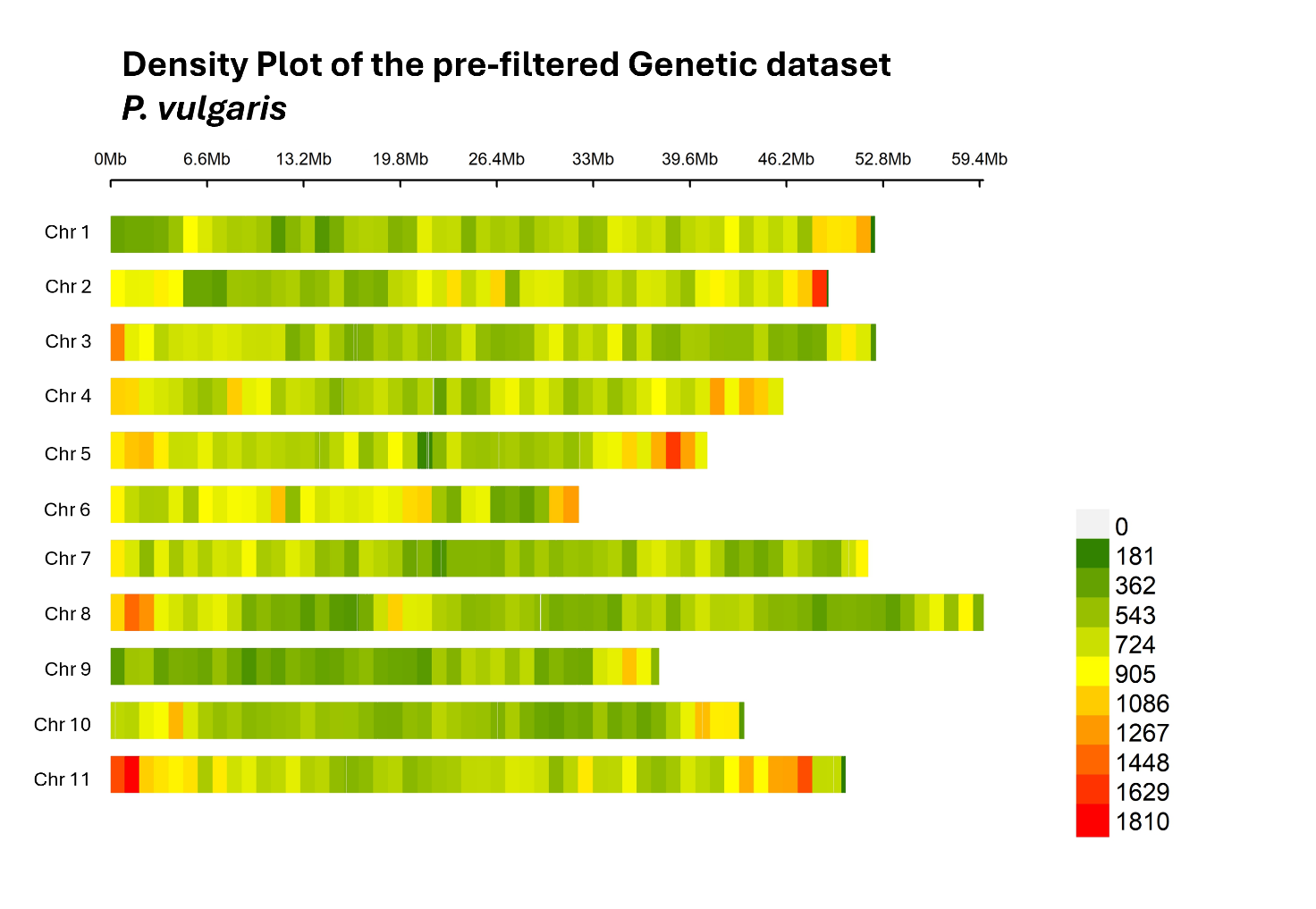


**Figure S2**: SNP density plot across the 11 chromosomes of *Phaseolus vulgaris* L. representing number of SNPs within 1 Mb window size. The horizontal axis represents the chromosome length in Mb. Different colors correspond to SNP density.


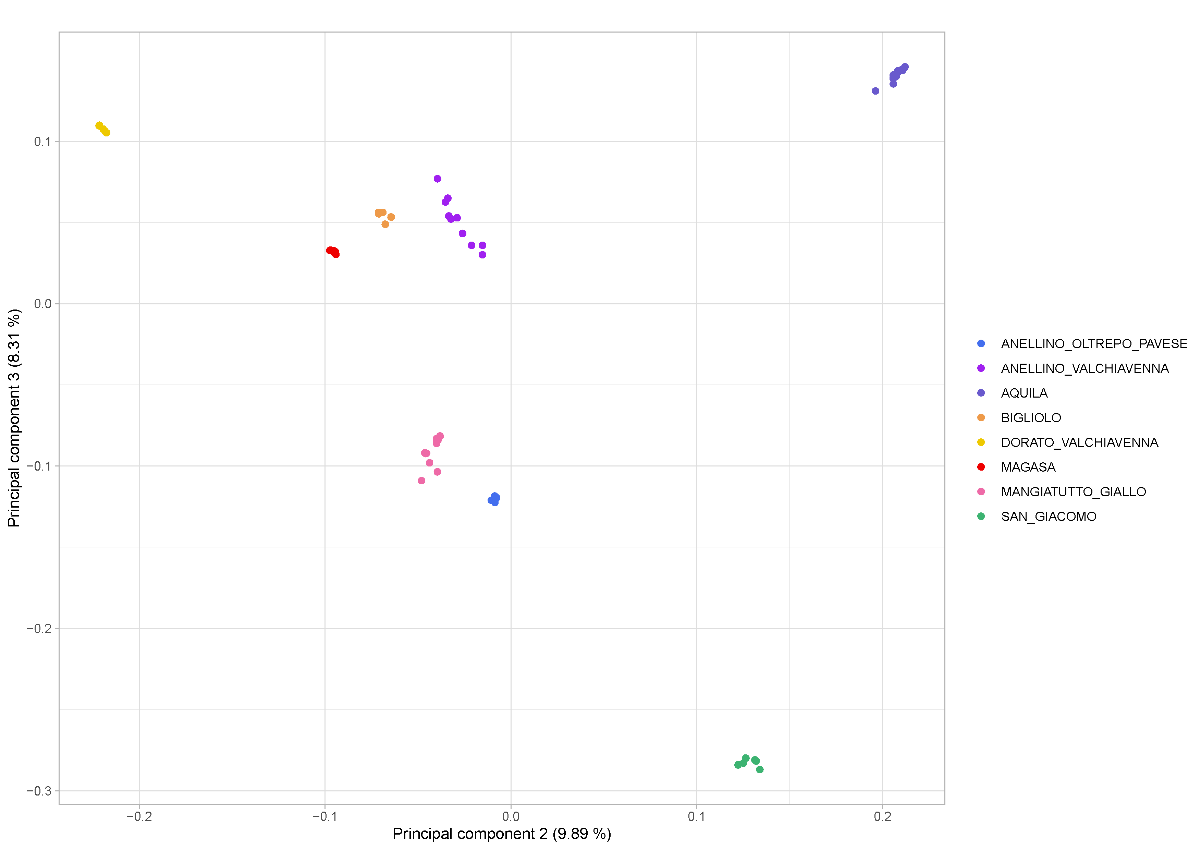


**A)**


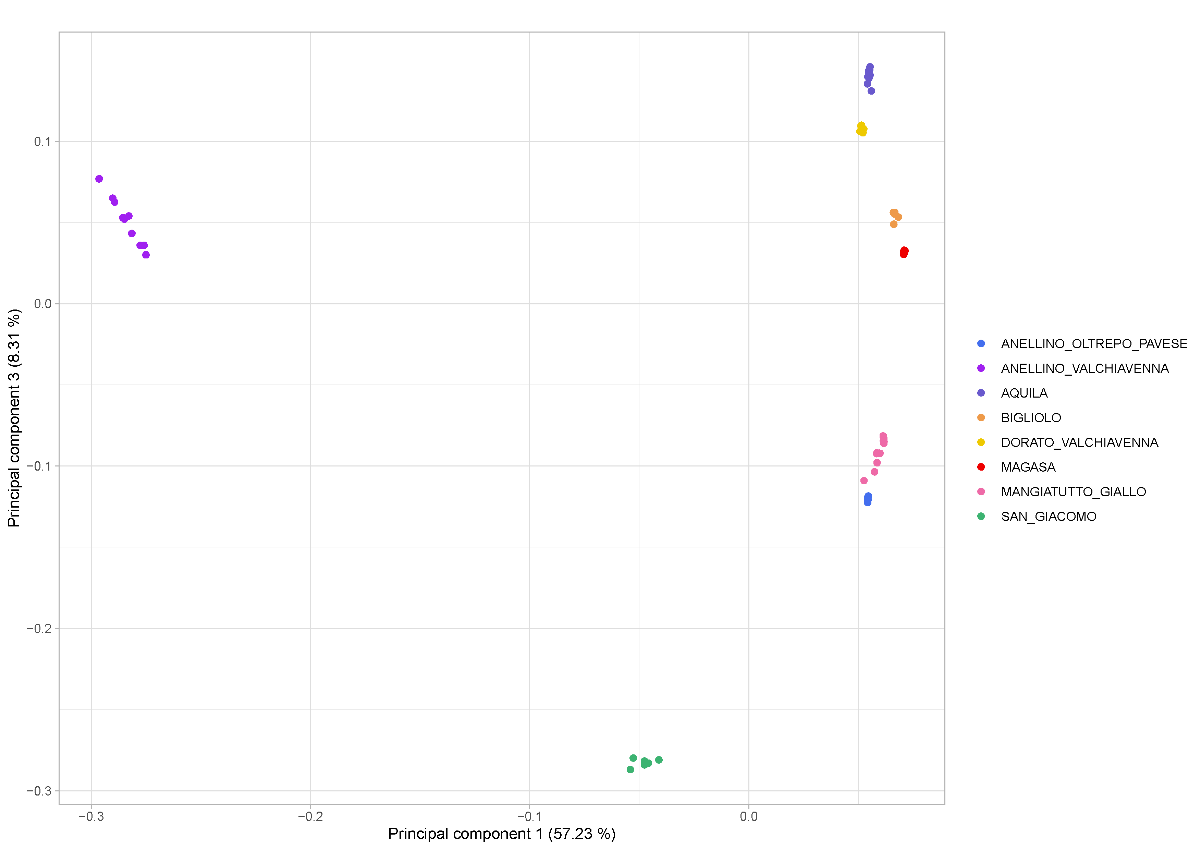


**B)**

**Figure S3**: principal component analysis of 65 samples of *Phaseolus vulgaris* L. landraces from Italy; the 16,622 SNPs set was used; A) Component 2 vs. Component 3; B) Component 3 vs. Component 1. ​


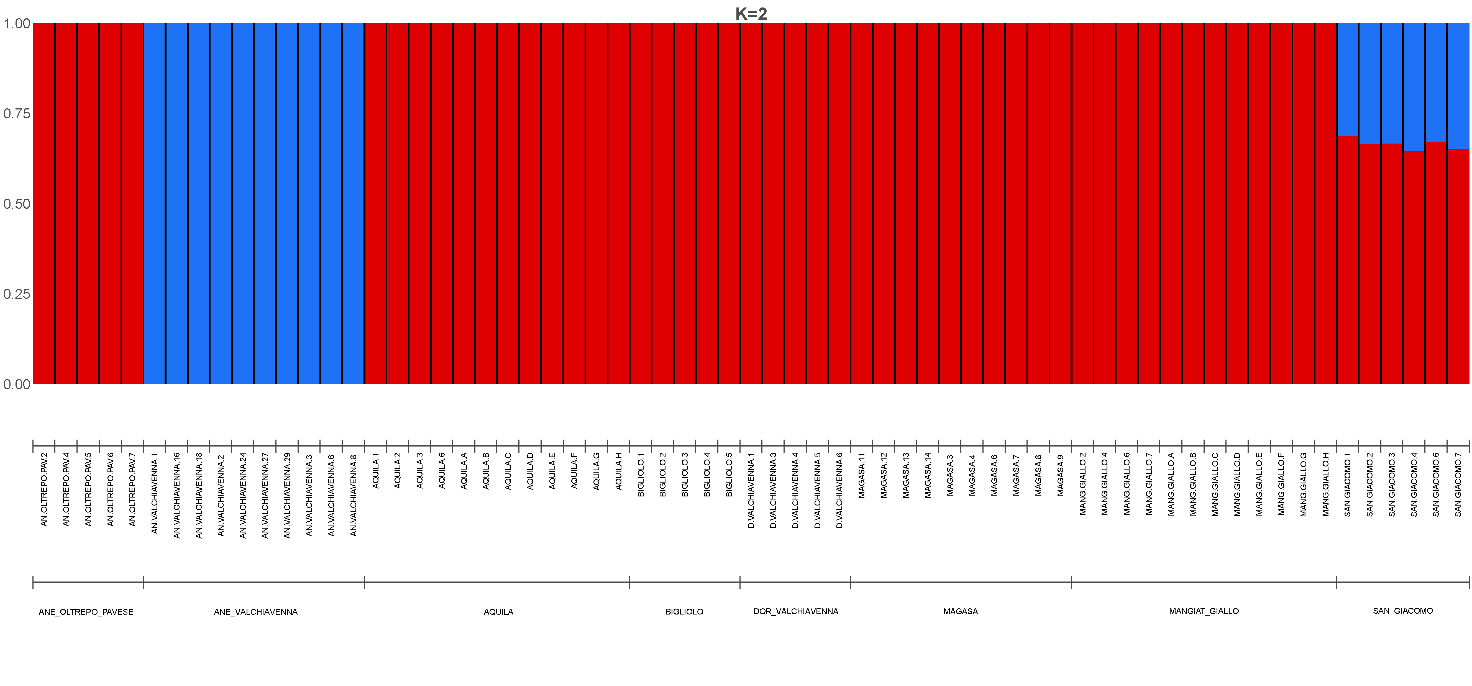

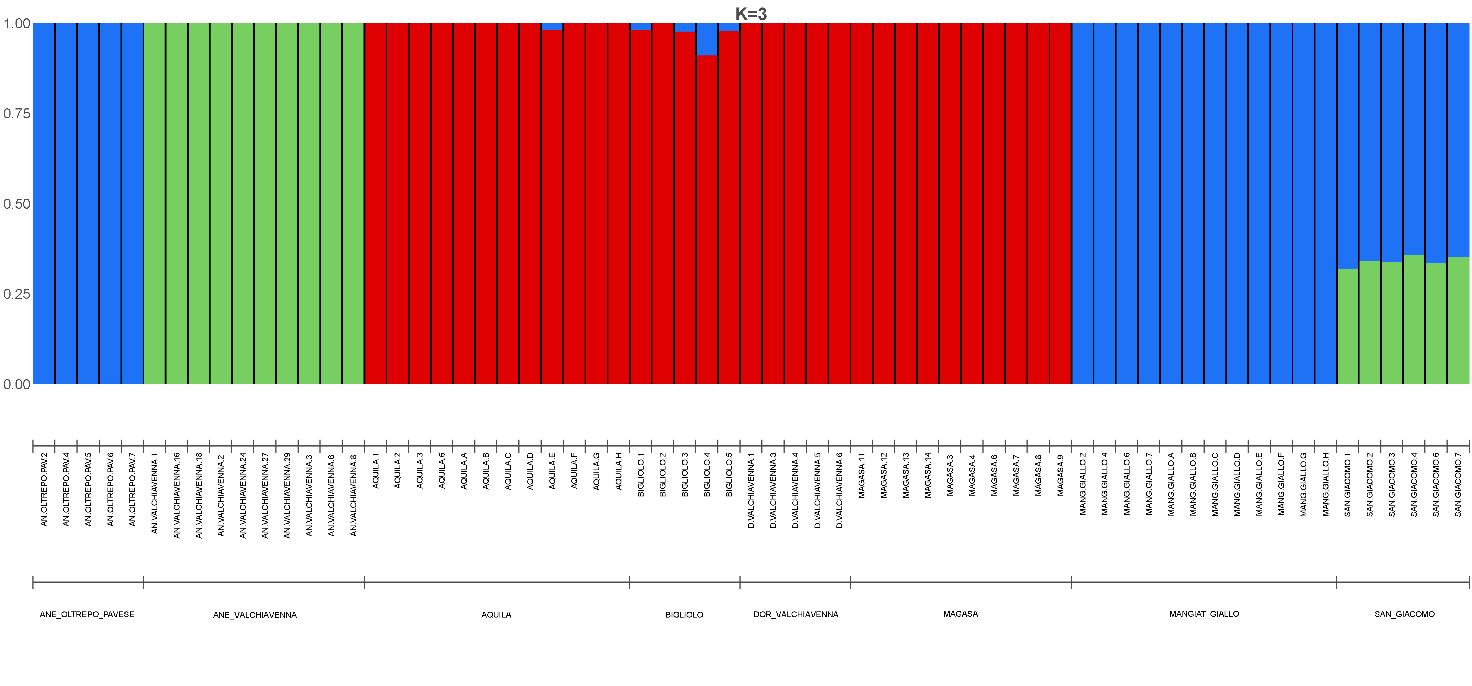

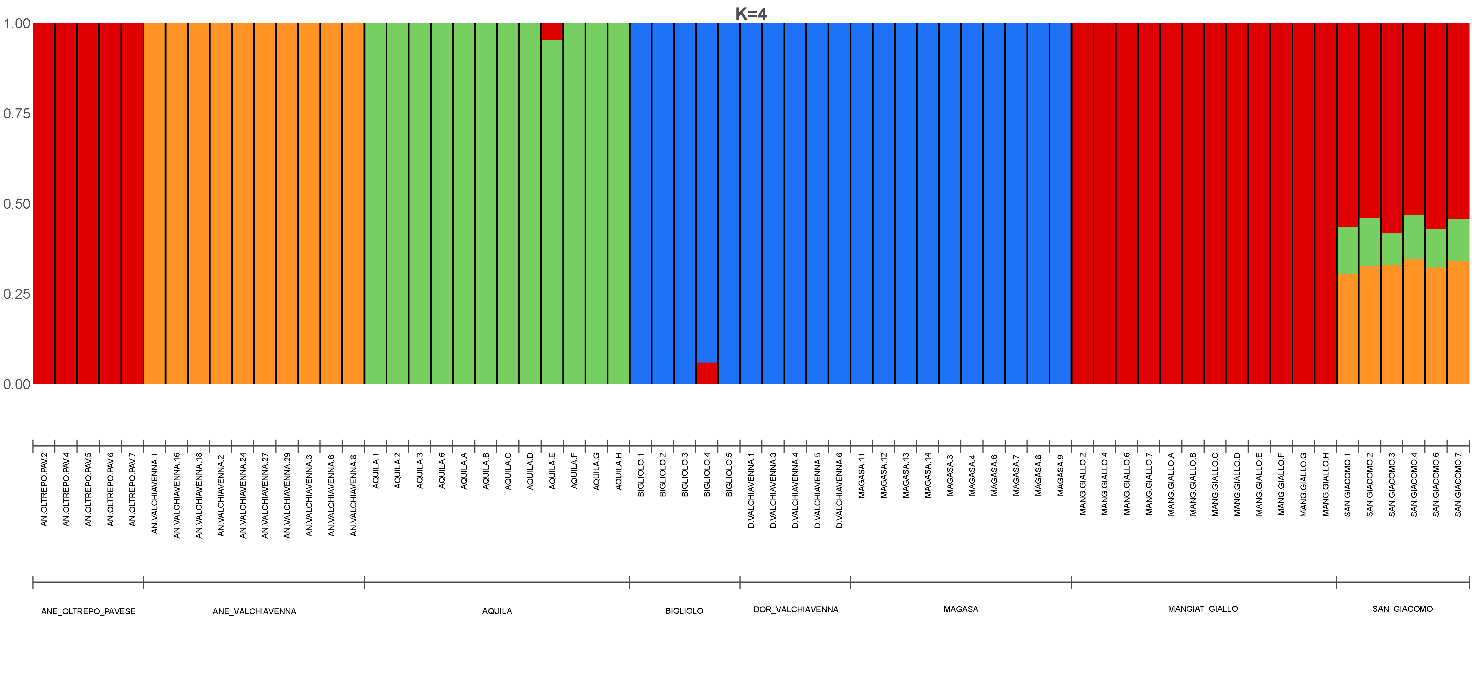

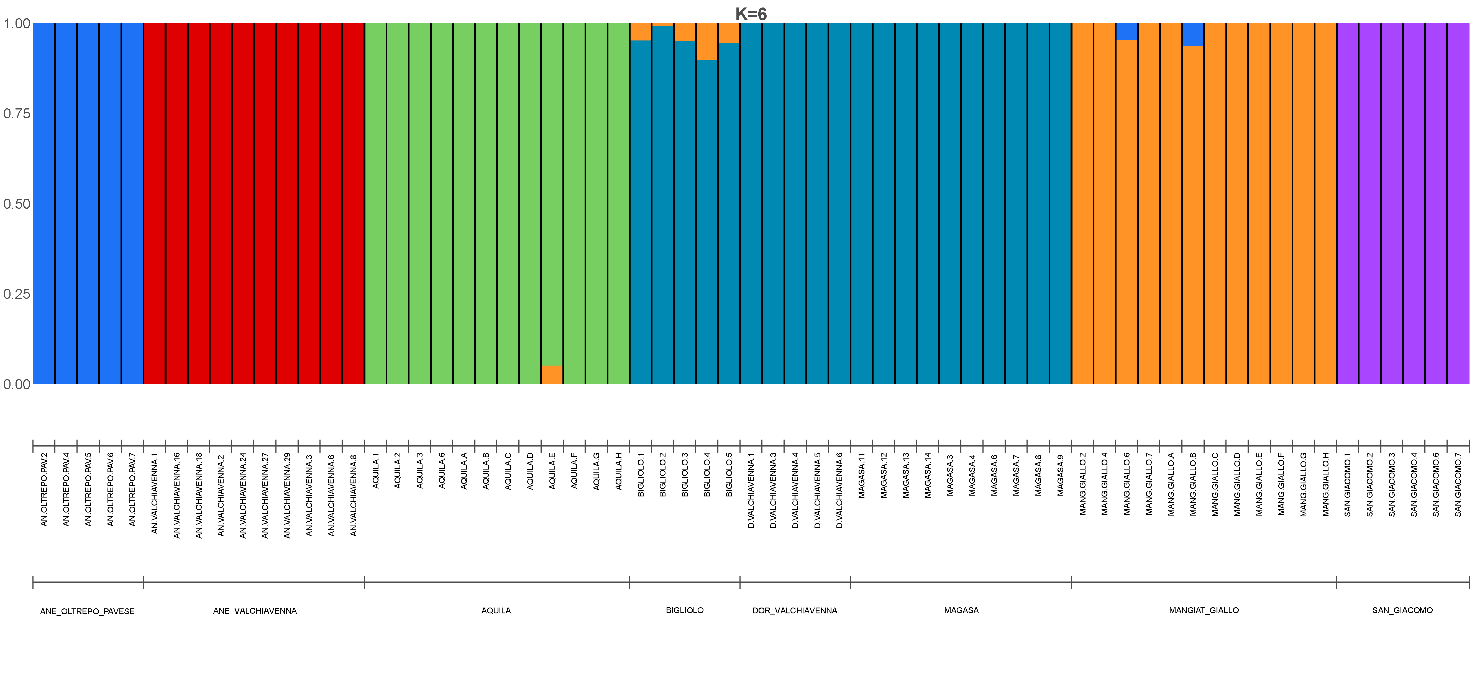
**Figure S4**: Population genetic structure at K = 2 (A); K = 3 (B); K = 4 (C); and K = 6 (D) of the 65 individuals of 8 *Phaseolus vulgaris* L. accessions evaluated in the present study. Different colours correspond to different ancestral populations.

**D)**

**C)**

**B)**

**A)**


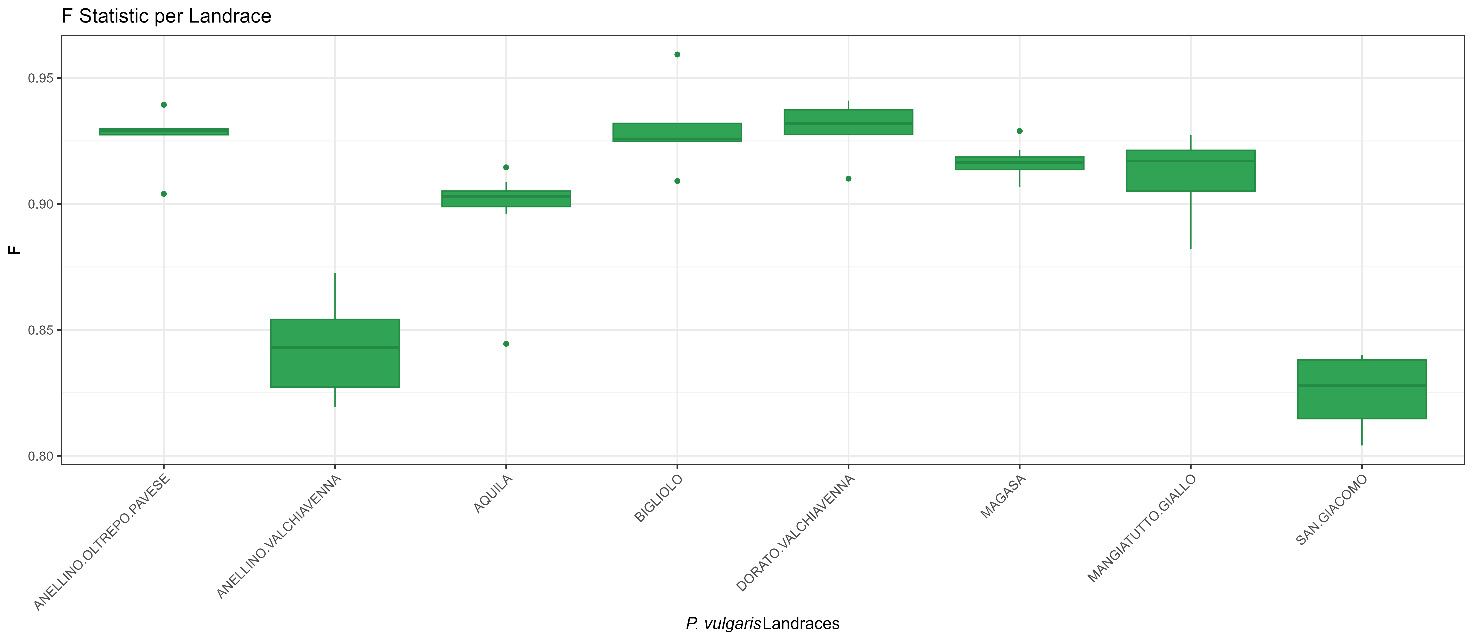


**Figure S5**: Boxplot of the inbreeding coefficient (F) computed for each of the filtered SNP and grouped by *P. vulgaris* landrace.


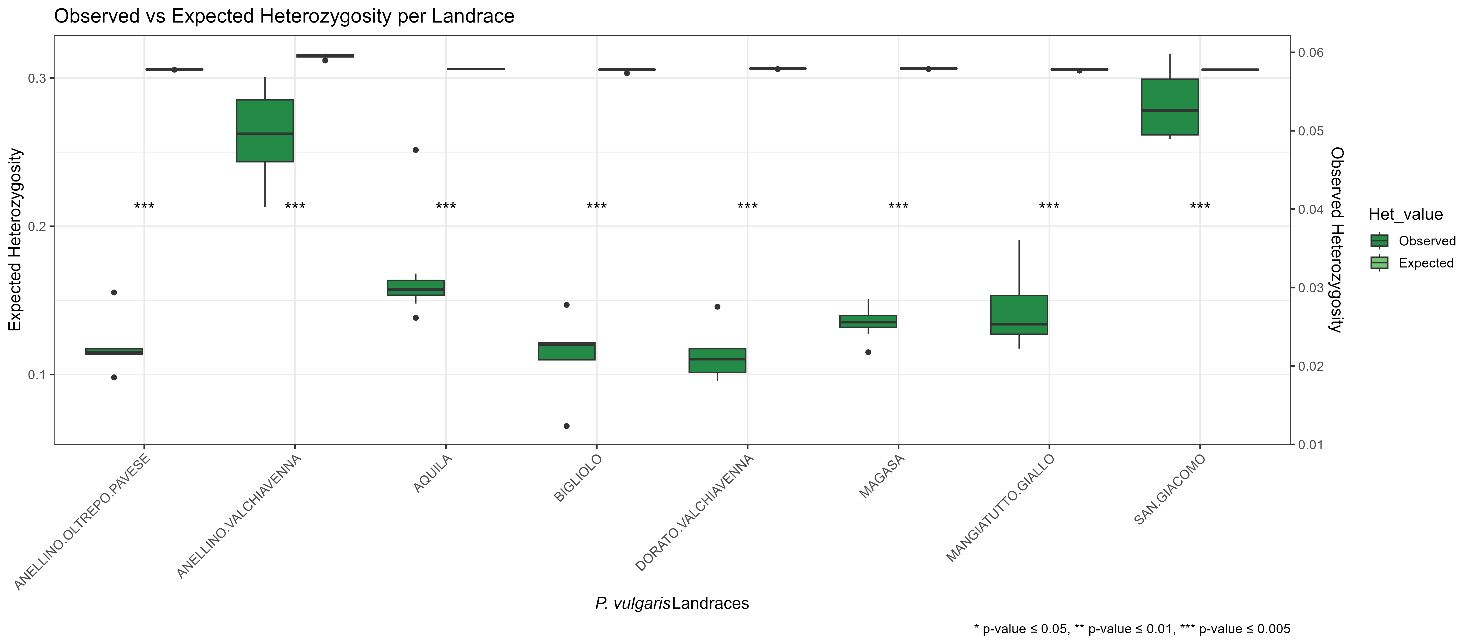


**Figure S6**: Comparison between Expected and Observed heterozygosity for each *P. vulgaris* landrace


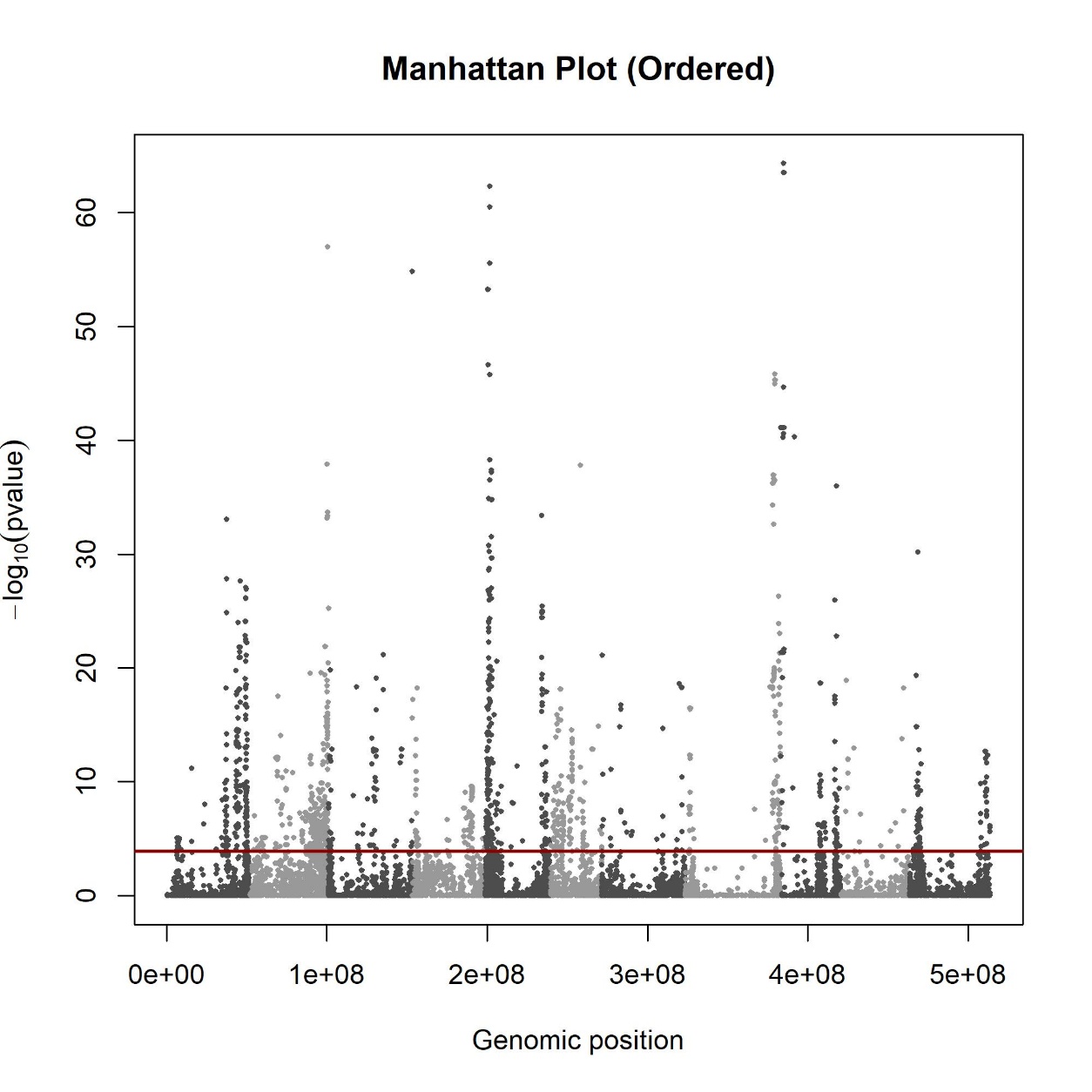


**Figure S7**: Manhattan plot of genome-wide SNP associations identified by pcadapt (K = 8) in Phaseolus vulgaris. Each point represents a SNP plotted as −log₁₀(p-value) against genomic position. The red line indicates the FDR significance threshold (α = 0.001).

**
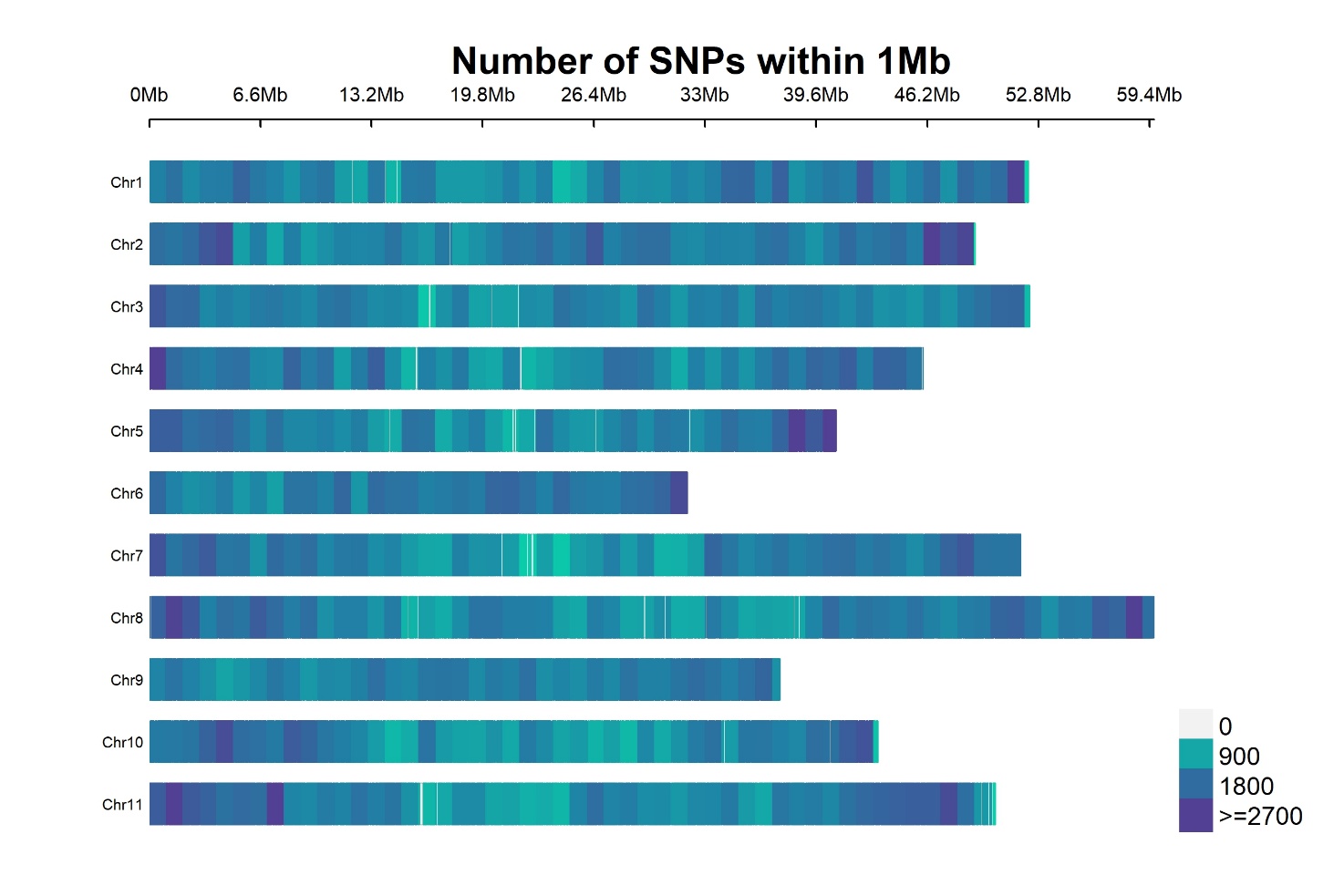
**

**Figure S8**: SNP density plot of *Phaseolus coccineus* L. SNPs mapped onto the 11 chromosomes of the *Phaseolus vulgaris* L. reference genome, showing the number of variants within 1-Mb windows. The horizontal axis represents chromosome length (Mb), and colors indicate SNP density.


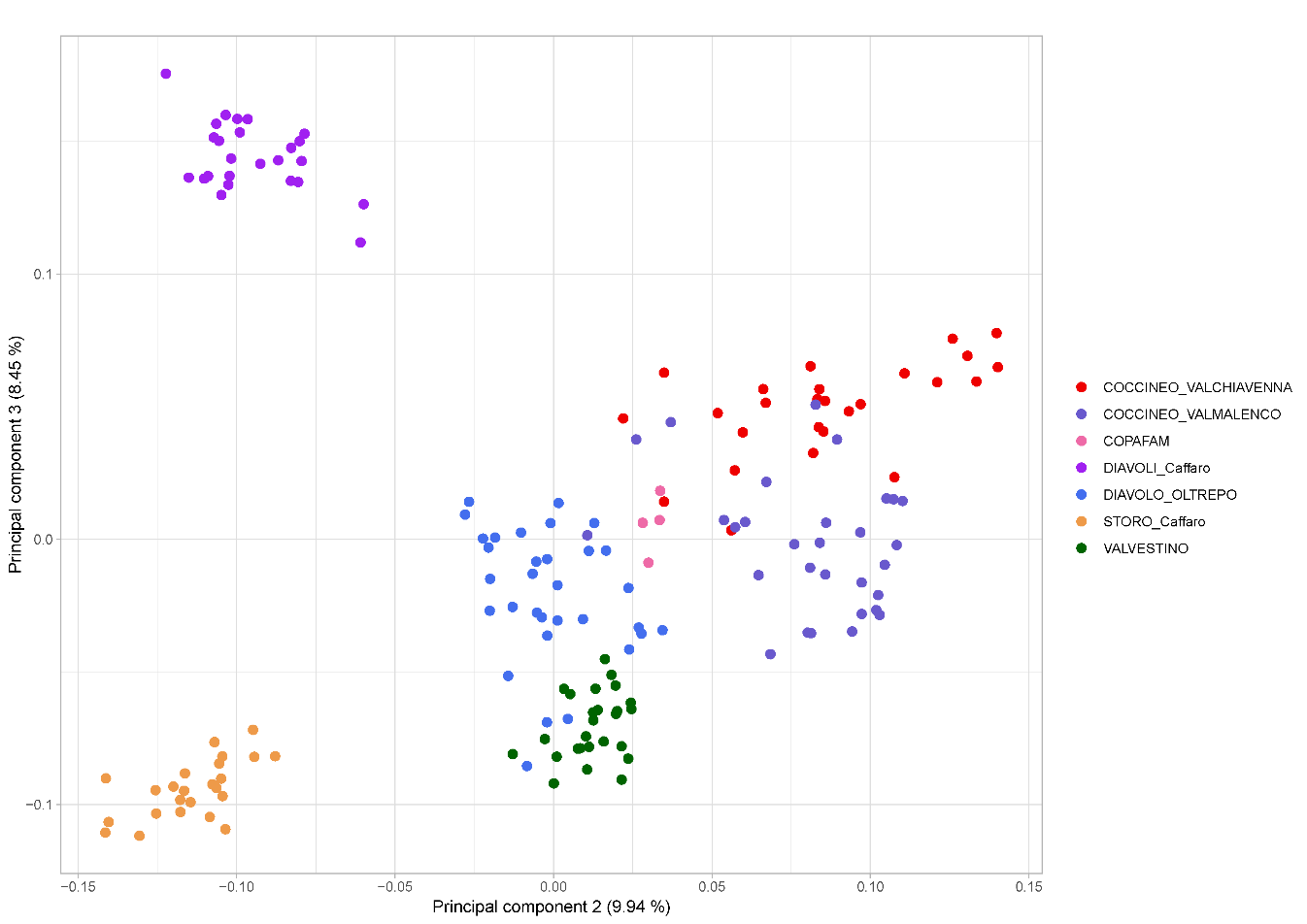

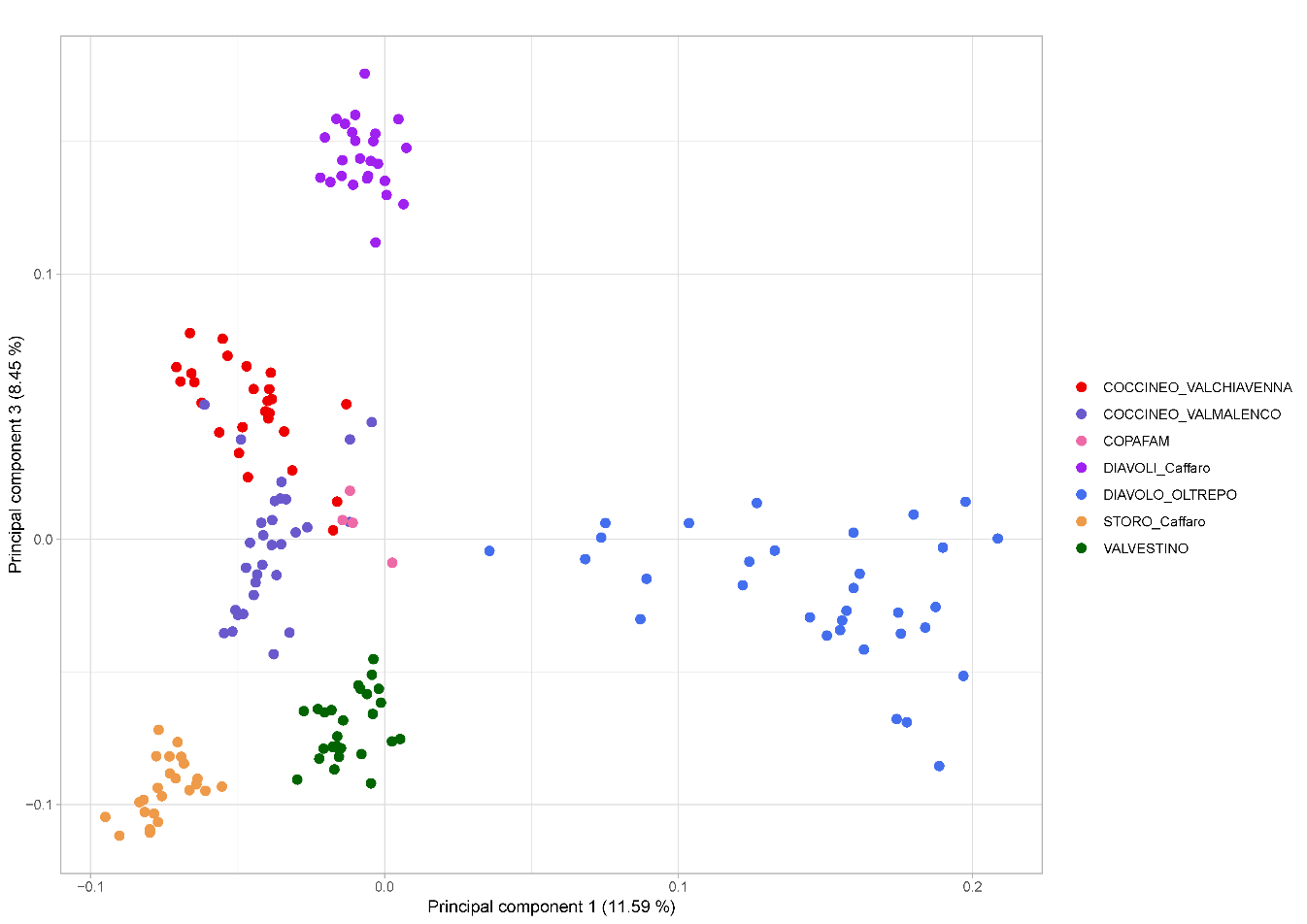


**B)**

**A)**

**Figure S9:** principal component analysis of 168 samples of *Phaseolus coccineus* L. landraces from Italy; the 28,006 SNPs set was used; A) Component 2 vs. Component 3; B) Component 3 vs. Component 1.


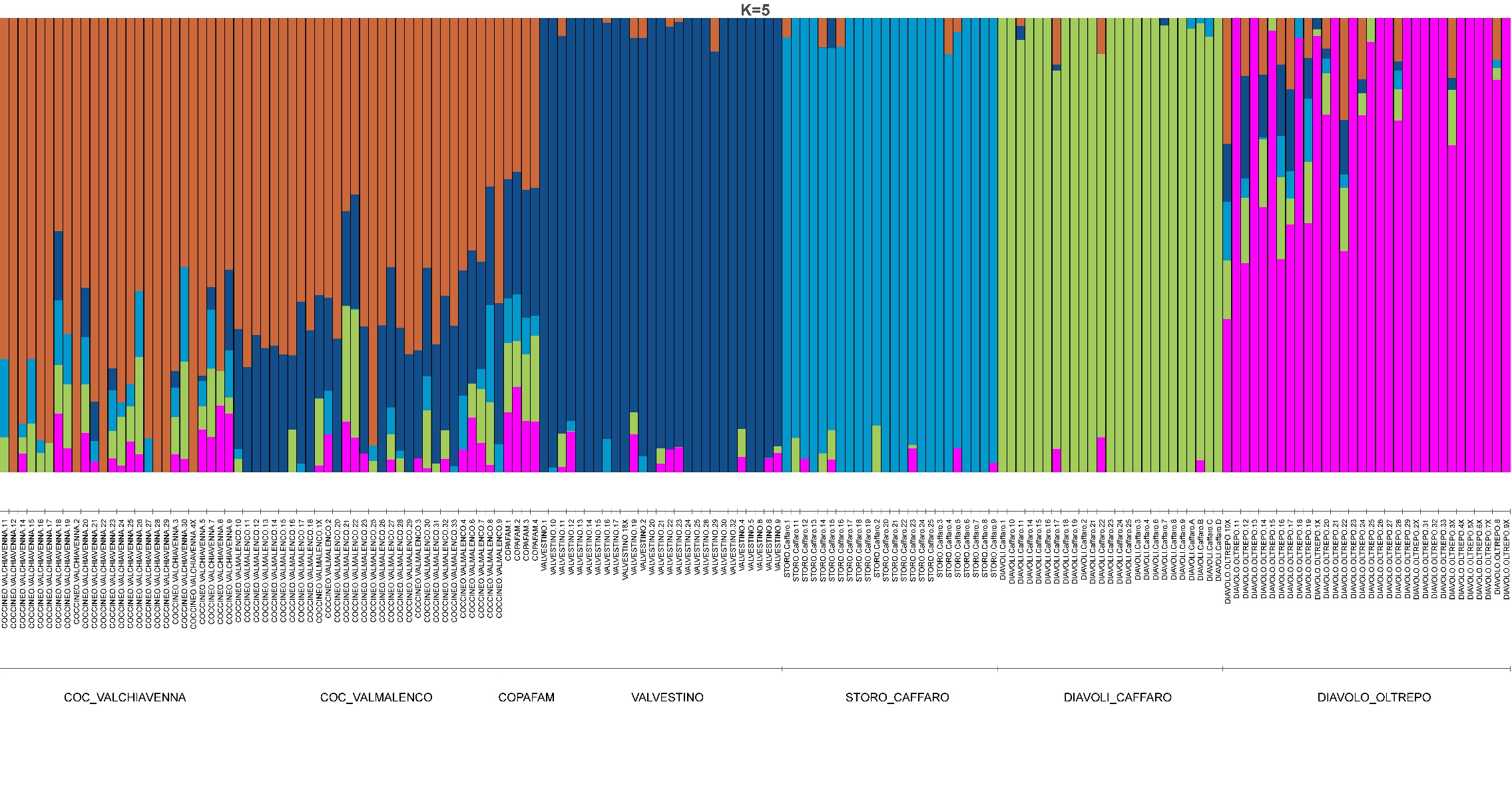


**A)**


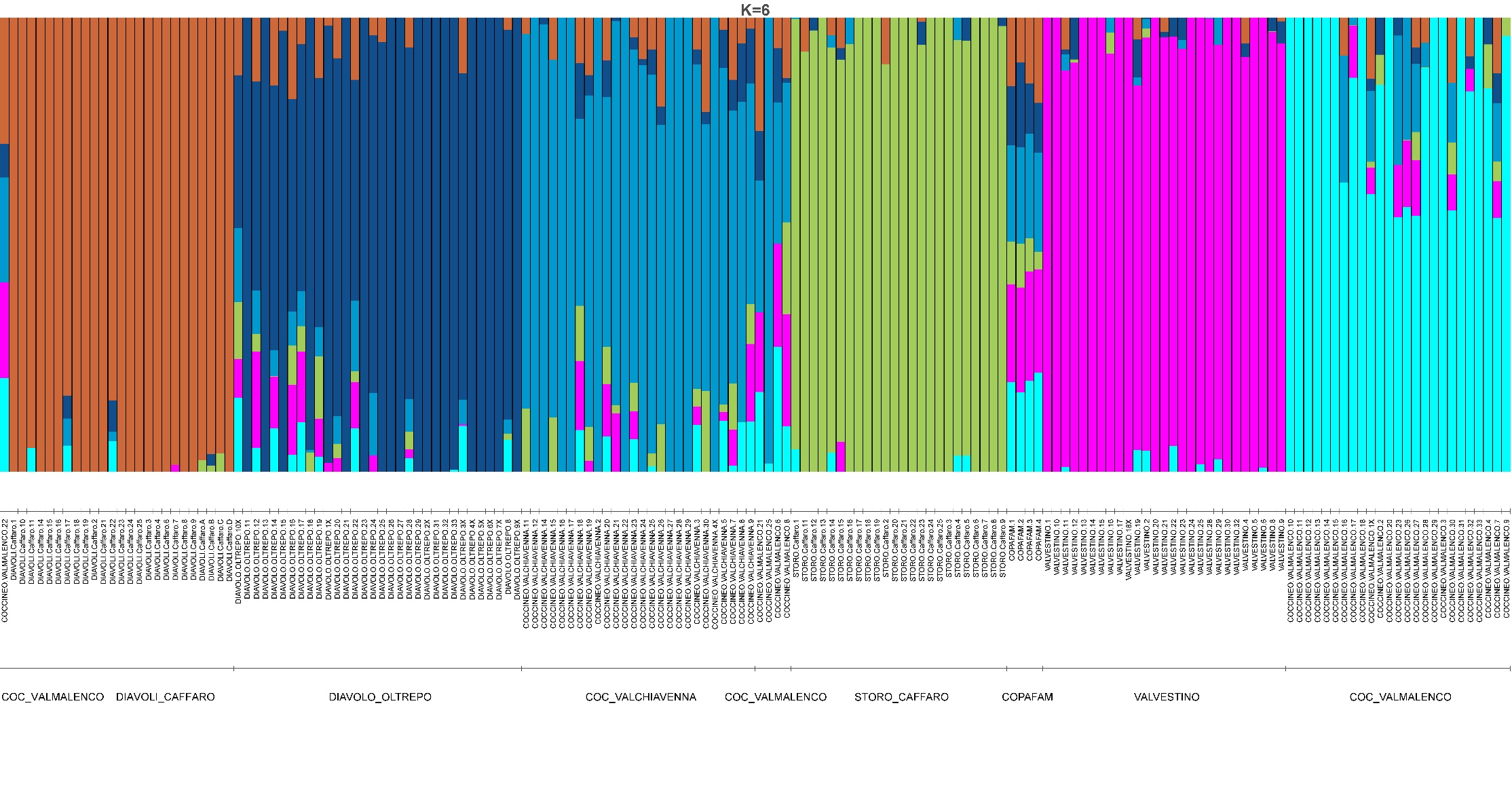


**B)**

**Figure S10**: Population genetic structure at K = 5 (A) and K = 6 (B) of the 168 individuals of 7 *Phaseolus coccineus* L. accessions evaluated in the present study. Different colors correspond to different ancestral populations.


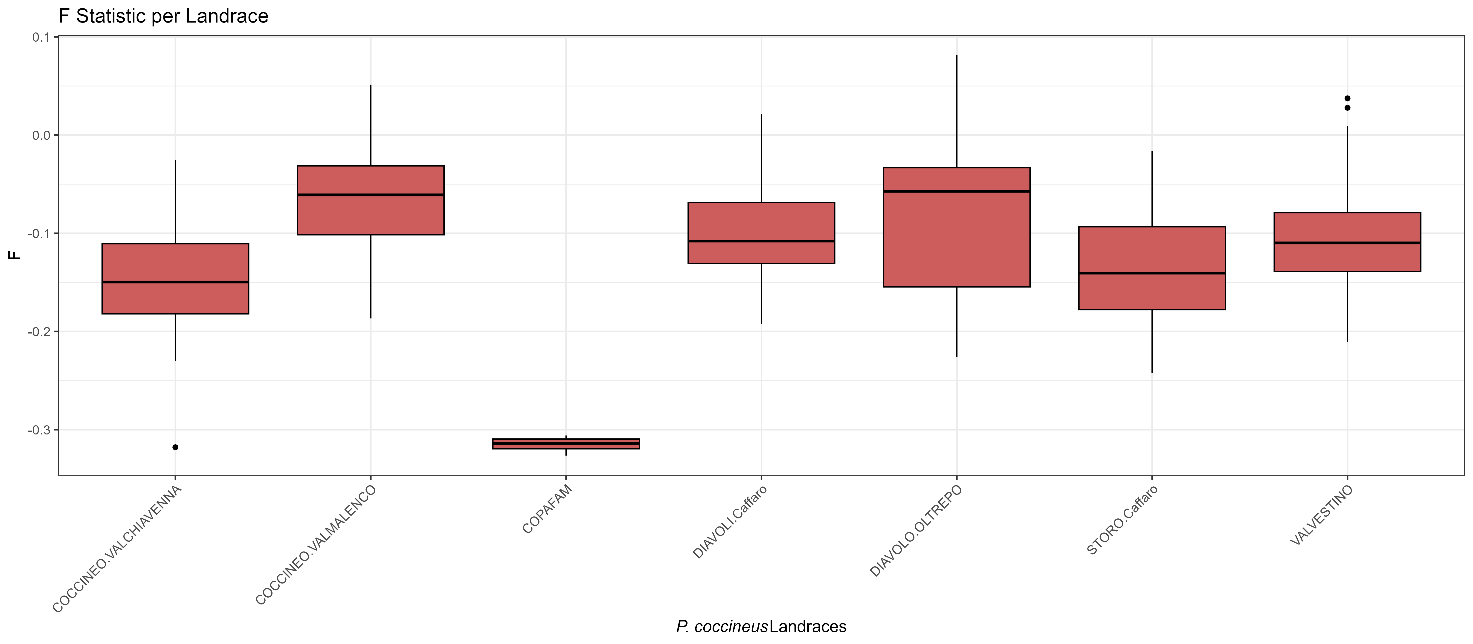


**Figure S11**: Boxplot of the inbreeding coefficient (F) computed for each of the filtered SNP and grouped by *P. coccineus* landrace.

**
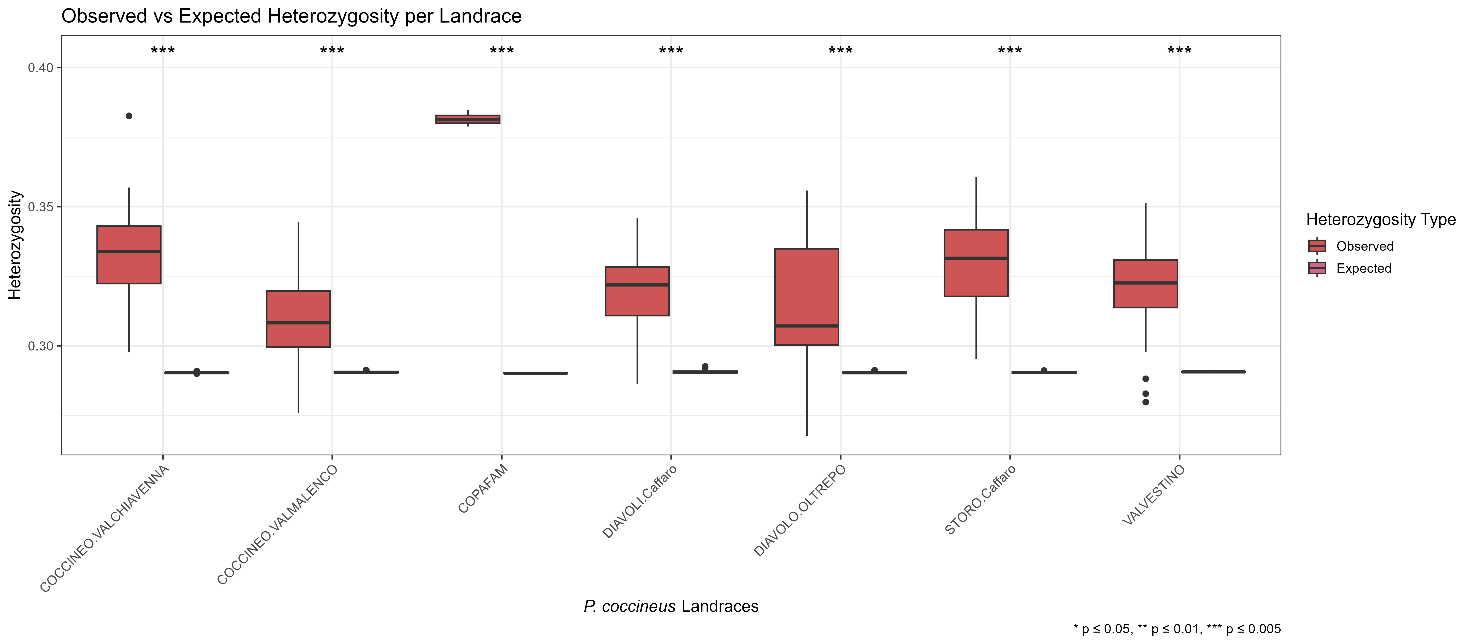
**

**Figure S12**: Comparison between Expected and Observed heterozygosity for each *P. coccineus* landrace.


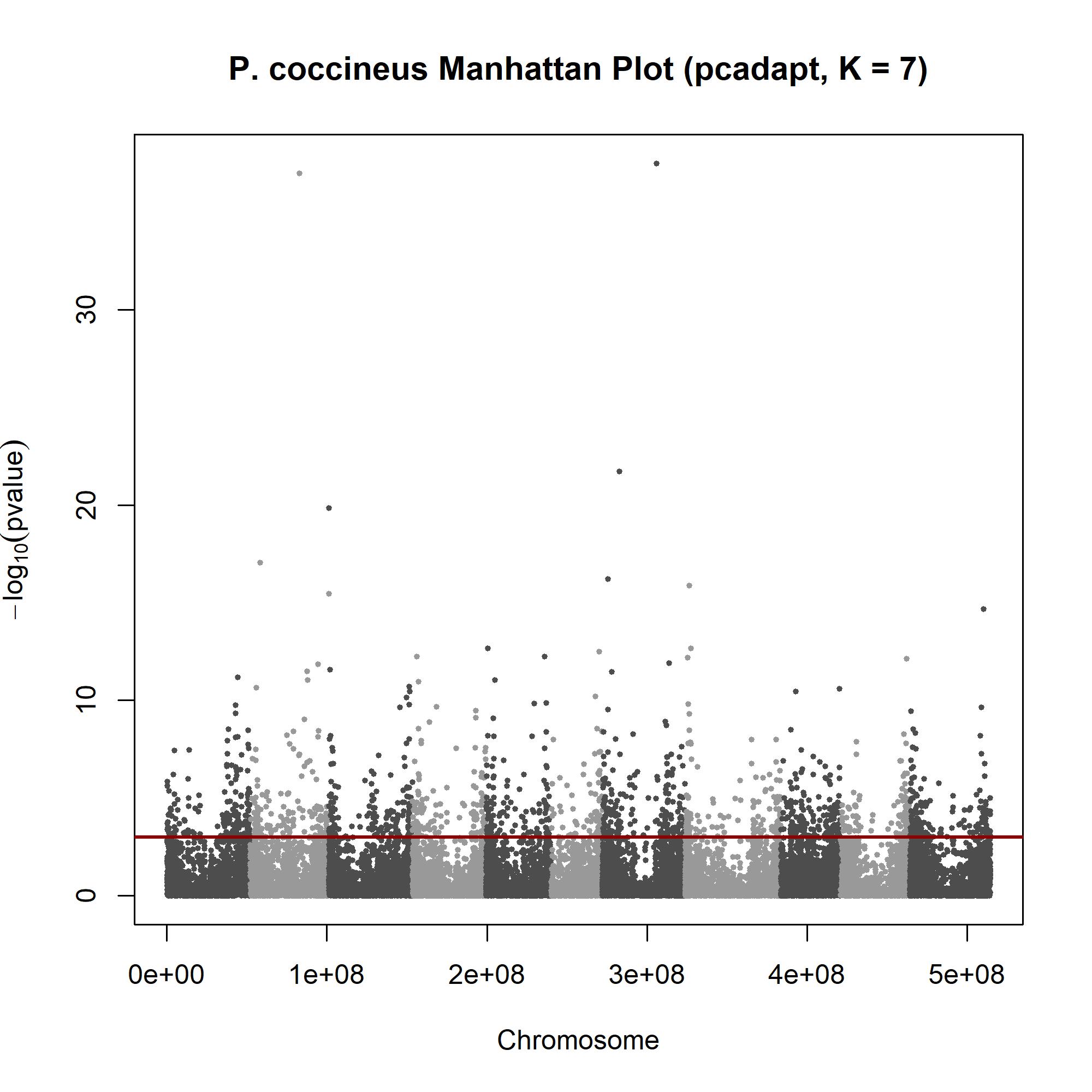


**Figure S13**: Manhattan plot of genome-wide SNP associations identified by *pcadapt* (K = 7) in *Phaseolus coccineus*. Each point represents a SNP plotted as −log₁₀(p-value) against genomic position. The red line indicates the FDR significance threshold (α = 0.001).
